# Effects of *Lactobacillus salivarius* isolated from feces of fast-growing pigs on intestinal microbiota and morphology of suckling piglets

**DOI:** 10.1038/s41598-021-85630-7

**Published:** 2021-03-24

**Authors:** Joseph Moturi, Kwang Yeol Kim, Abdolreza Hosseindoust, Jun Hyung Lee, Biao Xuan, Jongbin Park, Eun Bae Kim, Jin Soo Kim, Byung Jo Chae

**Affiliations:** 1 Department of Animal Industry Convergence, Kangwon National University, Chuncheon, 24341 Republic of Korea; 2 Department of Bio-Health Convergence, Kangwon National University, Chuncheon, 24341 Republic of Korea; 3 Poultry Research Institute, National Institute of Animal Science, Pyeongchang, 25342 Republic of Korea; 4 Department of Animal Resource Science, College of Animal Life Science, Kangwon National University, Chuncheon, 24341 Republic of Korea; 5 Department of Animal Biosciences, University of Guelph, Guelph, ON N1G 2W1 Canada; 6 Department of Applied Animal Science, College of Animal Life Science, Kangwon National University, Chuncheon, Kangwon-do Republic of Korea

**Keywords:** Biotechnology, Microbiology

## Abstract

The study determined the effects of *Lactobacillus salivarius* (LS) administered early in the life of suckling piglets on their growth performance, gut morphology, and gut microbiota. Thirty litters of 3-day-old crossbreed piglets were randomly assigned to one of the three treatments, and treatments were commenced on day 3 after birth. During the whole period of the experiment, the piglets were kept with their mothers and left to suckle ad libitum while being supplemented with a milk formula with or without the bacterial probiotic supplemented. The control group (CON) was not treated with probiotics, the HLS group was treated with LS144 (HLS) screened from feces of fast-growing pigs with high body mass index (BMI) while the NLS group was supplemented with LS160 (NLS) screened from feces obtained from pigs of normal BMI. At the weaning time, a higher abundance of Actinobacteria, Lentisphaerae, and Elusimicrobia phyla were observed in NLS piglets, whereas the abundance of Fibrobacteres phylum was significantly reduced in NLS and HLS piglets compared with the CON. A greater abundance of *Lactobacillus* was detected in the HLS treatment compared with the CON. The abundance of *Bacteroides* and *Fibrobacter* was higher in the CON piglets compared with the HLS and NLS piglets. Compared with the CON group, the oral administration of LS significantly increased the number of *Lactobacillus* and villus height in the duodenum, jejunum, and ileum. Moreover, the villus height of the duodenum was significantly improved in the HLS treatment compared with the NLS treatment. Based on the findings in the neonatal piglet model, we suggest that oral supplementation of LS, particularly LS isolated from high BMI pigs, could be beneficial by improving the intestinal villus height.

## Introduction

In swine, weaning and suckling are by far the most stressful periods that imposes the highest rate of loss and mortality. The adverse effect of diarrhea is more critical in suckling and weanling pigs than mature pigs due to the immature immune system^1,2^. A serious pathogenic challenge or stress during this critical neonatal period impacts negatively on the piglets whole process of development^2–4^. Therefore, the management of gut microbiota of suckling pigs by controlling *Clostridium* and *Escherichia coli* colonization may efficiently reduce the economic loss^2,3^. The microbiota in the small intestine is a dynamic ecosystem with a diverse commensal bacterial population, which affects the immune development and health of piglets^5–8^. Piglets are born with basically a sterile gut and the colonization begins immediately after birth^3,9^. In addition, the intestinal tract of neonatal piglets is under influence of undefined factors such as mother's feces and environmental microbes^2,3^, particularly that suckling piglets eat about 20 g feces per day due to their suckling habit^10^. Therefore, regarding the immature and unstable gut microbiota, any environmental stressors or pathogenic challenges may quickly compromise the microbiota equilibrium and compromise suckling pig health conditions.

After the ban on antibiotic growth promoters (AGP), probiotics have been found to be one of the most suitable alternatives to replace the AGPs in the animal industry as growth promoters. Many strains of bacteria have been tested for use as probiotics including *L. salivarius* SGL19, *Bacillus subtilis* B2A, *Lactobacillus acidophilus* K31*,* and *Enterococcus* M74^11–14^. During the suckling period, milk as the main feed source is regarded to be the most effective factor in shaping the intestinal microbiota of neonatal piglets. Among the beneficial genus, *Lactobacillus* spp. can be considered as one of the best candidates due to their high proliferation rate when milk or milk products are used as substrates^8^. It has been shown that *L. salivarius* is able to trigger the growth of the population of *Lactobacillus* spp. bacteria and decrease the colonization of pathogens due to their great ability to adhere to intestinal epithelial cells and produce bacteriocins^15–17^. *L. salivarius* is a Gram-positive bacteria and one of the major inhabitant of pigs’ intestine that is tolerant of acidic conditions with an optimal pH range of 5.5–6.5^17,18^. Moreover, in a recent study, *L. salivarius* exhibited activity against pathogenic bacteria such as Clostridia, *Campylobacter*, and *Salmonella* in both in vivo and in vitro^18–21^. Consequently, dietary supplementation of *L. salivarius* appears to be beneficial to the pig gut health by influencing intestinal gut microbial colonization.

In recent years, high-throughput sequencing platforms such as 16S rRNA gene amplicon sequencing is extensively being applied to reveal the community structures of the microbiota. It is reported that there is an interaction between the intestinal microbiota and body weight in pigs^15,22,23^, particularly in young animals due to the immature intestinal microbial community. In the current study, after the screening process of potential *Lactobacillus* sp. with high bile and acid tolerance, antimicrobial activity, and adhesion capacity, the *L. salivarius* (LS144) from the feces of fast-growing pigs was detected to be used for further analysis. In addition, as a control treatment, *L. salivarius* (LS160) from normal weight pigs was isolated through the same procedure. Regarding our in vitro tests, we hypothesized that the two targeted strains of *L. salivarius* have diverse influences on the microbial proportion of Firmicutes to Bacteroidetes. This in vivo study was undertaken to investigate the effects of *L. salivarius* (LS144 and LS160) on weight gain, intestinal microorganism composition, and intestinal histomorphology of suckling pigs.

## Results

### Microbial community structure

**Figure 1:**
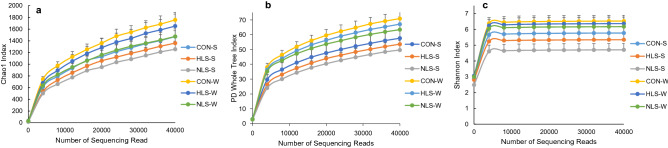
Diversity of intestinal microbiota of piglets at different stages. Alpha diversity indices including Chao1 (**a**), PD whole tree (**b**), and Shannon (**c**) were observed at each number of sequencing reads.

**Figure 2:**
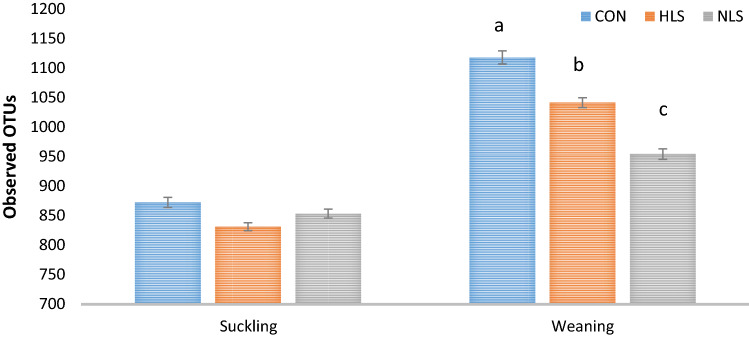
OTUs gain at the beginning (Suckling) and the end (weaning) of the experiment. CON, Control without probiotic; HLS, *L. salivarius* 144 isolated from fast-growing pig feces; NLS, *L. salivarius*160 isolated from normal weight pig feces and different superscript letters indicate significant differences (P < 0.05).

**Figure 3:**
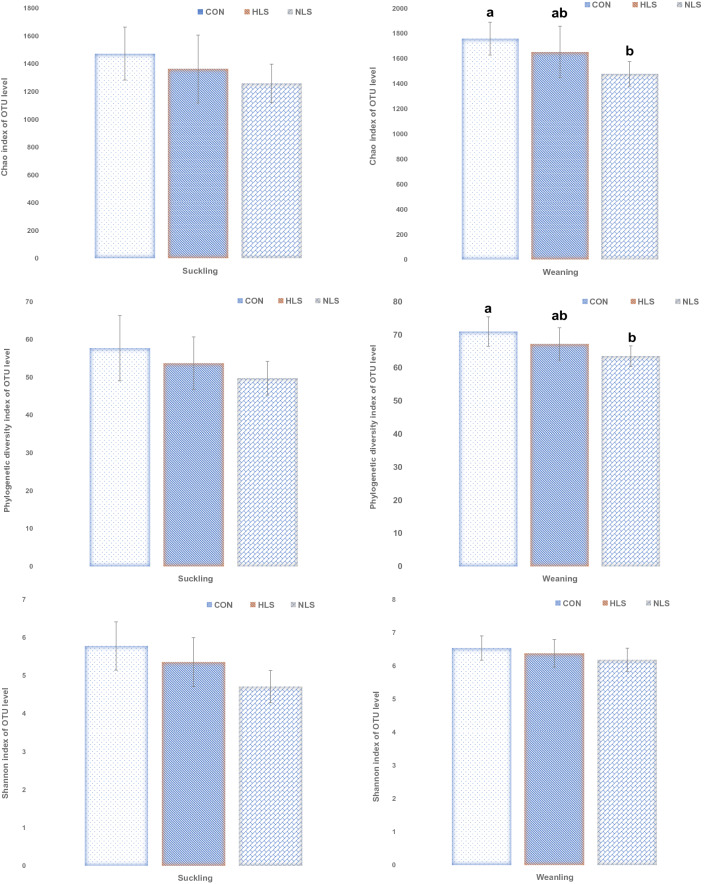
Differences in the fecal microbial species richness and diversity indices (Chao 1, Shannon, OTU; ≥ 97% sequence similarity threshold) per treatment. Different superscript letters indicate significant differences (P < 0.05).

**Figure 4:**
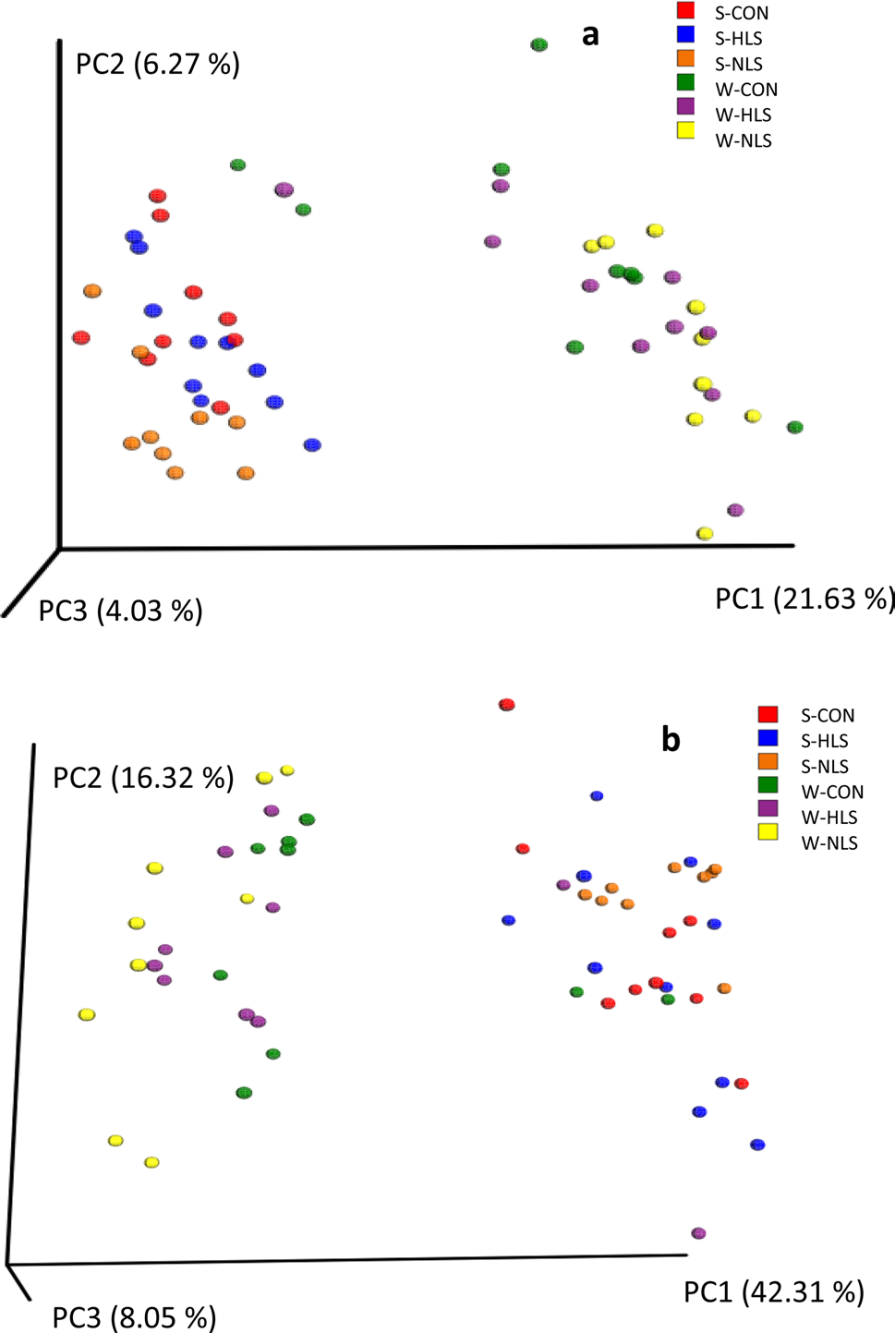
Beta diversity patterns of fecal microbial diversity in at the beginning (d 4, suckling) and end of the experiment (d 21, weaning) as assessed by principal coordinate analysis (PCoA) of unweighted and Weighted Unifrac. CON, Control without probiotic; HLS, *L. salivarius* 144 isolated from fast-growing pig feces; NLS, *L. salivarius*160 isolated from normal weight pig feces.

An average of 40,000 16S rRNA gene sequence reads was generated (Fig. 1). The number of observed OTUs (± SE) was 872.4 (± 19.3) for the CON group, 831.4 (± 18.5) for the HLS group (LS isolated from the feces of fast-growing pigs), and 853.6 (± 10.8) for the NLS (LS isolated from the feces of normal weight pigs) group at suckling period (Fig. 2). At weaning, the OTU value was 1117.3 (± 9.0) for the CON group, 1040.8 (± 11.5) for the HLS group, and 953.9 (± 7.9) for the NLS group. During the sucking phase, there was no difference in microbiota diversity (Fig. 3). However, a significant (p = 0.002) decrease in the Chao index (Fig. 3), which reflects the species evenness and richness, was observed in the NLS treatment compared with the CON at weaning. At weaning time, a higher (p = 0.005) phylogenetic diversity index was observed in pigs in the CON treatment compared with the NLS treatment. No difference in the Shannon index was detected between the treatments. The Adonis test for the analysis of similarities of unweighted UniFrac distances (Fig. 4a) indicated no difference between the treatments, however, there was a significant difference (R^2^ = 0.14, P < 0.01) between suckling and weaning time, showing that the microbiota of piglets was significantly changed over the time. There was a similar analysis of similarities between weighted UniFrac distances and unweighted UniFrac distances (Fig. 4b), which showed no difference among the treatments but a distinct clustering (R^2^ = 0.25, P < 0.01) between sucking and weaning times.

### Taxa difference at the phylum level

**Figure 5:**
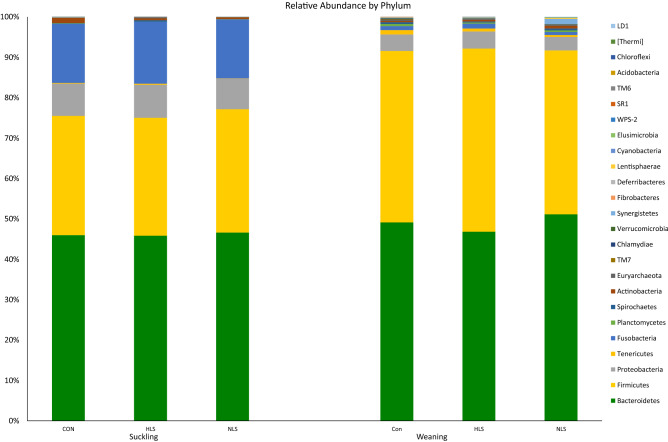
16S rRNA gene analysis revealed the relative abundance of fecal bacterial community structure at the phylum level in piglets orally treated with probiotics *Lactobacillus salivarius* 144 (HLS), *L. salivarius* 160 (NLS), or without probiotic (CON) at suckling (d 4) and weaning (d 21) periods.

At the 97% similarity level, in total 25 phyla (Fig. 5) were detected. At the suckling period, the two dominant phyla detected in the three groups were Bacteroidetes (45.9%) and Firmicutes (29.8%). The analysis of microbiota in piglets showed a higher abundance of Firmicutes, Tenericutes, Lentisphaerae, Deferribacteres, Elusimicrobia, and Fibrobacteres phyla, and a lower abundance of Fusobacteria, Proteobacteria, and Actinobacteria in the weaning period compared with the suckling period (Table 1). At the weaning period, again Bacteroidetes (49.0%) and Firmicutes (42.8%) were the dominant phyla. A higher abundance of Actinobacteria, Lentisphaerae, and Elusimicrobia phyla were observed in NLS piglets, whereas the abundance of Fibrobacteres phylum was significantly reduced in NLS and HLS piglets compared with the CON.

**Table 1:** Relative abundance of the fecal microbiota phyla of three groups at suckling (d 4) and weaning (d 21) periods.

	Suckling			Weaning			P-value		
	CON	HLS	NLS	CON	HLS	NLS	Suckling	Weaning	Time
Bacteroidetes	45.98 ± 7.9	44.61 ± 10.1	46.33 ± 11.3	49.15 ± 9.6	46.83 ± 9.3	51.14 ± 3.1	0.61	0.12	0.69
Firmicutes	29.69 ± 6.0	29.23 ± 14.2	30.42 ± 4.4	42.42 ± 9.2	45.35 ± 9.5	40.59 ± 5.1	0.48	0.23	0.00
Fusobacteria	14.63 ± 7.4	15.35 ± 9.7	14.60 ± 12.3	0.99 ± 0.61	1.20 ± 0.95	0.74 ± 0.61	0.78	0.21	0.00
Proteobacteria	9.033 ± 5.3	9.169 ± 4.8	7.724 ± 4.5	4.101 ± 0.7	4.229 ± 1.8	3.341 ± 0.8	0.52	0.12	0.00
Actinobacteria	1.276 ± 1.0	0.489 ± 0.28	0.654 ± 0.27	0.31 ± 0.15^b^	0.40 ± 0.31^ab^	0.67 ± 0.37^a^	0.13	0.02	0.15
Tenericutes	0.143 ± 0.21	0.269 ± 0.71	0.048 ± 0.02	1.080 ± 0.93	0.739 ± 0.63	0.469 ± 0.23	0.33	0.06	0.00
Planctomycetes	0.120 ± 0.22	0.012 ± 0.02	0.063 ± 0.003	0.517 ± 0.41	0.276 ± 0.21	0.406 ± 0.36	0.11	0.13	0.00
Lentisphaerae	0.042 ± 0.08	0.006 ± 0.01	0.007 ± 0.009	0.032 ± 0.02^b^	0.086 ± 0.11^ab^	0.216 ± 0.30^a^	0.11	0.05	0.01
Deferribacteres	0.002 ± 0.004	0.0001 ± .0004	0.0002 ± .0001	0.072 ± 0.13	0.017 ± 0.02	0.032 ± 0.03	0.17	0.14	0.01
Elusimicrobia	0.004 ± 0.003	0.001 ± 0.001	0.007 ± 0.006	0.017 ± 0.01^b^	0.009 ± 0.007^b^	0.114 ± 0.176^a^	0.14	0.03	0.03
Fibrobacteres	0.004 ± 0.004	0.002 ± 0.002	0.015 ± 0.0038	0.073 ± 0.069^a^	0.016 ± 0.022^b^	0.017 ± 0.023^b^	0.22	0.01	0.00

CON, Control; HLS, *Lactobacilus salivarius* 144 isolated from fast-growing pig feces; NLS, *L. salivarius*160 isolated from normal weight pig feces.

^a-b^Means with different superscripts within rows are significantly different at P < 0.05.

### Taxa difference at the genus level

**Figure 6:**
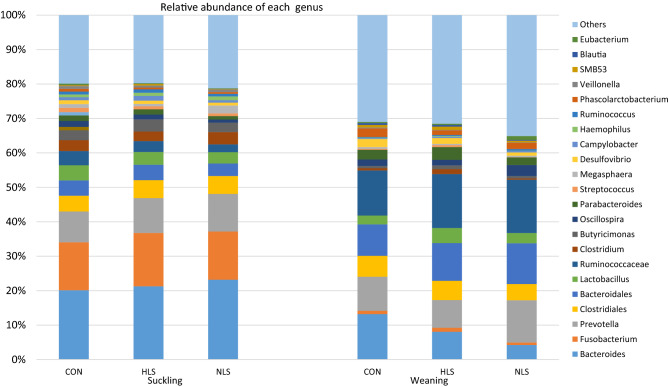
16S rRNA gene analysis revealed the relative abundance of fecal bacterial community structure at the genus level in piglets orally treated with probiotics *Lactobacillus salivarius* 144 (HLS), *L. salivarius* 160 (NLS), or without probiotic (CON) at suckling (d 4) and weaning (d 21) periods.

At the 97% similarity level, in total 450 genera (Fig. 6) were detected. At the genus level, three dominant genera, *Bacteroides* (21.69%), *Fusobacterium* (14.91%), and *Prevotella* (10.01%) were detected in the fecal microbiota of piglets at the suckling period, whereas microbiota of weaned piglets was dominated by *Prevotella* (10.07%), *Bacteroides* (8.47%), and *Lactobacillus* (3.31%). At weaning time, although the differences in the abundance of *Lactobacillus* did not differ between the HLS and NLS piglets, a significantly greater *Lactobacillus* population was recorded in the HLS treatment compared with the CON (Table 2). The abundance of *Bacteroides* and *Fibrobacter* was higher in the CON piglets compared with the HLS and NLS piglets (Table 2). Compared to the CON, the abundance of *Phascolarctobacterium* was lower in the HSL, and the abundance of *Desulfovibrio*, *Clostridium*, and *Weissella* was lower in the NLS treatment. The highest abundance of *Christensenella* and *Limnohabitans* genera were observed in the HLS treatment, whereas the highest abundance of *Helicobacter* and *Methanosphaera* was detected in the NLS treatment. The piglets fed HLS probiotic showed a lower abundance of *Oscillospira*, and a greater abundance of *Bacteroides*, *Sarcina*, *Limnohabitans*, and *Christensenella* compared with the NLS treatment.

**Table 2:** Relative abundance of the fecal microbiota genera of three groups at weaning (d 21).

Item	Treatment			P-value
	CON	HLS	NLS	
*Bacteroides*	13.19 ± 4.7^a^	8.04 ± 3.3^b^	4.20 ± 3.1^c^	0.041
*Prevotella*	9.86 ± 6.9	8.06 ± 7.7	12.30 ± 6.1	0.428
*Ruminococcus*	1.57 ± 1.1	1.80 ± 1.1	1.17 ± 1.1	0.494
*Lactobacillus*	2.58 ± 1.4^b^	4.40 ± 2.3^a^	2.97 ± 2.4^ab^	0.046
*Parabacteroides*	2.81 ± 1.6	3.72 ± 1.6	2.22 ± 1.6	0.139
*Oscillospira*	1.90 ± 1.3^ab^	1.61 ± 0.65^b^	3.21 ± 1.7^a^	0.028
*Phascolarctobacterium*	2.50 ± 1.5^a^	1.32 ± 1.1^b^	1.81 ± 1.4^ab^	0.010
*Desulfovibrio*	2.29 ± 0.86^a^	1.69 ± 0.86^ab^	0.96 ± 0.53^b^	< 0.01
*Campylobacter*	0.241 ± 0.21	0.359 ± 0.50	0.444 ± 0.45	0.589
*Streptococcus*	0.249 ± 0.18	0.552 ± 0.13	0.060 ± 0.025	0.308
*Clostridium*	1.437 ± 1.38^a^	0.616 ± 0.38^ab^	0.330 ± 0.30^b^	0.028
*Fusobacterium*	0.983 ± 0.61	1.192 ± 0.95	0.741 ± 0.61	0.438
*Fibrobacter*	0.074 ± 0.07^a^	0.016 ± 0.0^b^	0.018 ± 0.0^b^	0.010
*Helicobacter*	0.029 ± 0.02^b^	0.043 ± 0.02^b^	0.265 ± 0.18^a^	< 0.01
*Bifidobacterium*	0.014 ± 0.01	0.013 ± 0.02	0.026 ± 0.02	0.244
*Christensenella*	0.007 ± 0.001^b^	0.021 ± 0.01^a^	0.005 ± 0.003^b^	0.020
*Sarcina*	0.004 ± 0.004^ab^	0.015 ± 0.01^a^	0.003 ± 0.002^b^	0.027
*Weissella*	0.003 ± 0.003^a^	0.001 ± 0.001^ab^	0.0003 ± 0.0003^b^	0.014
*Limnohabitans*	0.002 ± 0.002^b^	0.024 ± 0.019^a^	0.001 ± 0.001^b^	0.011
*Methanosphaera*	0.001 ± 0.001^b^	0.0001 ± 0.00003^b^	0.004 ± 0.003^a^	< 0.01

CON, Control; HLS, *Lactobacilus salivarius* 144 isolated from fast-growing pig feces; NLS, *L. salivarius*160 isolated from normal weight pig feces.

^a-b^Means with different superscripts within rows are significantly different at P < 0.05.

### Intestinal digesta microbial population

**Figure 7:**
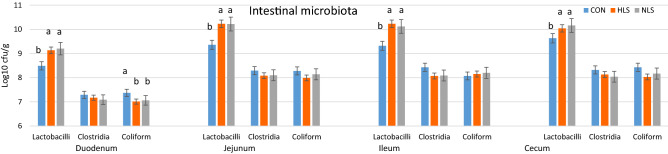
The population of lactobacilli, clostridia, and coliform at different sections of the intestine at the end of study (d21, weaning). CON, Control without probiotic; HLS, *L. salivarius* 144 isolated from fast-growing pig feces; NLS, *L. salivarius*160 isolated from normal weight pig feces.

Intestinal digesta analysis revealed a significant increase of *Lactobacillus* in the duodenum, jejunum, ileum, and cecum of pigs fed HLS and NLS *Lactobacillus* (P < 0.01). The total number of coliforms was significantly reduced in the duodenum of pigs in the HLS and NLS treatments compared with the CON treatment (P < 0.05), however, there were no differences in the population of coliforms in the jejunum, ileum, and cecum. There was no significant difference in the colonization of Clostridia among the treatments in all the segments of the intestine (Fig. 7).

### Intestinal morphology

Both HLS and NLS treatment groups had significantly increased (P < 0.01) villi height throughout the 3 segments of the intestinal tract (duodenum, jejunum, and ileum) as compared with the CON group (Table 3). The crypt depth did not differ between the groups in all the 3 intestinal sections. However, the villus height and crypt depth ratio (VH:CD) differed significantly (P < 0.01) among the groups in the ileum with the greatest value in the HLS treatment group and the lowest value in the CON group.

**Table 3:** Effects of dietary supplementation of *Lactobacillus salivarius* on intestinal gut morphology of piglets at weaning.

Item	Treatment			SEM	P-value
	CON	HLS	NLS		
Villus height (VH)					
Duodenum	257.07^c^	283.46^a^	269.03^b^	3.71	0.001
Jejunum	246.87^b^	278.16^a^	270.76^a^	4.67	0.002
Ileum	150.93^b^	176.73^a^	168.73^a^	3.70	0.001
Crypt depth (CD)					
Duodenum	139.99	147.96	148.41	2.22	0.238
Jejunum	138.73	147.1	145.69	1.98	0.19
Ileum	121.8	117.5	123.22	1.32	0.191
VH:CD					
Duodenum	1.84	1.92	1.82	0.03	0.486
Jejunum	1.78	1.89	1.86	0.02	0.243
Ileum	1.24^c^	1.51^a^	1.37^b^	0.03	0.001

CON, Control; HLS, *L. salivarius* 144 isolated from fast-growing pig feces; NLS, *L. salivarius*160 isolated from normal weight pig feces.

^a-b^ Means with different superscripts within rows are significantly different at P < 0.05.

### Weight gain

The effect of the HLS and NLS supplementation on piglet growth performance was shown in Table 4. Weight gain and ADG were not affected by the LS-treatment groups compared to the CON group.

**Table 4:** Effect of dietary supplementation of *Lactobacillus salivarius* on piglet growth performance.

Item	Treatment			SEM	P-value
	CON	HLS	NLS		
Initial BW, kg (3 days)	1.55	1.54	1.54	0.01	0.274
Finishing BW, kg (21 days)	6.19	6.23	6.24	0.08	0.974
ADG, g	221.05	223.07	223.45	3.85	0.969

CON, Control; HLS, *L. salivarius* 144 isolated from fast-growing pig feces; NLS, *L. salivarius*160 isolated from normal weight pig feces. BW, body weight; ADG, average daily gain.

## Discussion

Infancy is a critical period due to unstable gut microbiome structure^2,3^. Dietary supplementation of probiotic lactobacilli may modulate the microbial community in the gastrointestinal tract preventing diarrhea and stimulate growth^14,24,25^. This study was conducted to evaluate the effects of oral dietary supplementation of live *L. salivarius* suspension in suckling piglets concerning growth performance, intestinal bacterial diversity, and intestinal morphology. In the current study, we evaluated the fecal microbiota composition in piglets fed two different LS during the suckling period. The present study has revealed that the supplementation of NLS significantly decreased the observed OTUs than those for the CON group. The lower Phylogenetic diversity index and Chao index of feces bacteria in the NLS treatment suggested that probiotics may inhibit the growth of bacteria, which was consistent with Wang et al.^26^ who reported a lower bacterial diversity along with a promoted intestinal health when using *L. casei* ZX633. Several studies have reported that intestinal microbial richness is an index with a positive relation to body weight^23^. It has been known that the increased microbiota diversity is associated with a stable ecology and overall health of animals^27^. Despite the benefits, the abundance of microbiota may adversely affect the host in several ways such as immune system stimulation, nutrient competition, and the generation of toxic catabolites^9^.

In our study, the relative abundance of phyla in both LS-treated treatments were significantly changed compared with the CON. The microbiota of the NLS group showed a higher abundance of Actinobacteria, Lentisphaerae, and Elusimicrobia compared with that of the CON group, whereas a significantly higher Fibrobacteres level was observed in the CON group. The Fibrobacteres phylum is related to cellulolytic bacteria energy metabolism^28^, and the reasons for these significant differences are not clear. The diversity of major phyla including Bacteroidetes, Firmicutes, Fusobacteria, and Proteobacteria were unaffected by the HLS or NLS treatments. This is in agreement with the results reported in a previous study with no difference in the levels of the main phyla such as Firmicutes and Bacteroidetes when using probiotics^18^. Bacteroidetes and Firmicutes are the most abundant phyla in piglet intestinal microbiota, regardless of dietary probiotic and age^9,29^. In this study, the most abundant phyla were Bacteroidetes and Firmicutes accounted for less than 80% of the phyla at the suckling period, however, at weaning time both Bacteroidetes and Firmicutes showed to be the most abundant phyla (more than 90% of the phyla) in feces, which is in agreement with some earlier studies^29,30^. The abundance of Proteobacteria and Fusobacteria was dramatically reduced from the suckling period to the weaning period. Fusobacteria has the potential to be pathogenic and be related to cancer and some other diseases in animals^31^. Moreover, within the Proteobacteria phylum, there are some pathogenic genera such as *Salmonella*, *Escherichia*, and *Helicobacter*^32^. This change in microbial composition is related to age, physiological, and dietary factors^16,29^. As shown in our study, it can be suggested that pathobiont species are a part of normal microbiota in infants and any stressors such as changing the diet formulation or form (at weaning period) may trigger these potential pathogens to proliferate.

At the genus level, within *Bacteroides*, this study identified a higher relative abundance of *Bacteroides* in the CON pigs. Surprisingly, the population of *Bacteroides* in NLS pigs was lower than in HLS pigs. The genera *Bacteroides* and *Prevotella* were reported to be the normal inhabitant of the intestine in pigs and sows^29,30,32^ although their population in the fecal microbiota of suckling pigs was at a much lower abundance than a previous study^33^. *Bacteroides* are naturally mutualistic species in the intestine, however, some of them are opportunistic pathogens^34^. *Bacteroides fragilis* is a good example of a pathogenic *Bacteroides* with the potential of causing malignancy, inflammation, and diarrhea^35^. In this study, *Bacteroides* were the most abundant, and *Fusobacterium* and *Prevotella* remained less abundant microbiota, in contrast to a previous study^32^. The administered HLS probiotic increased the population of *Lactobacillus*, *Limnohabitans*, *Sarcina*, and *Rhodoferax*, which belong to the phyla *Firmicutes* and *Proteobacteria*. Moreover, *Fusocaterium* was the most prominent genus of the phylum *Fusobacteria* indigenous to the fecal microbiota of piglets in this study. A plethora of factors such as diet^8,36–38^ affects intestinal microbiota communities of the host. Interestingly, the microbiota analysis of feces indicates that there is a negative relationship between the abundance of *Lactobacillus* and *Clostridium*, as the supplementation of HLS decreased the population of *Clostridium* and increased the population of *Lactobacillus* in the feces. Clostridia species are normally known to be pathogenic^2,4,12^. As there was a significant increase in the abundance of fecal *Lactobacillus* in HSL piglets, it is not surprising that a higher population of *Lactobacillus* was detected in the jejunum, ileum, and cecum of piglets fed LS. *Lactobacillus* species are considered to be among the beneficial members of a normal microbiota^5,7,22^. In this study, the most evident response of using *L. salivarius* seems to be the stimulating role in the growth of intestinal *Lactobacillus*, irrespective of its strain. Despite the increase in the population of *Lactobacillus* in the jejunum, ileum, and cecum of piglets in NLS treatment, the total abundance of *Lactobacillus* in the microbiota of feces was not affected. Whereas piglets in the HLS group not only showed an increased population of *Lactobacillus* in the jejunum, ileum, cecum but also revealed a greater abundance of fecal *Lactobacillus.* An increase in the abundance of *Lactobacillus* during the suckling period is essential to encounter the weaning time when piglets are highly vulnerable to opportunistic pathogens. The decrease in the proportion of fecal clostridia in piglets administered HLS or NLS when compared to the CON may highlight the antipathogenic effects on Gram-negative bacterium. Probiotics aid the host animal in defense against pathogens through competitive exclusion and the production of antimicrobials^5,12,39^. A similar study also reported that *L. salivarius* greatly increased the integrity of epithelial cells in pigs by protecting the small intestine cells from the adherence of enterotoxigenic *E. coli* K88, resulting in a higher survival rate^40^. However, our study does not show a straightforward confirmation of anti-pathogenic factors due to insignificant differences in the colonization of *Clostridium* and *E. coli* in the jejunum and ileum.

Improved villus height is a marker for better digestive and absorptive intestinal capacities^4,25^. In the current study, *L. salivarius* (LS144 and LS160) supplementation had a positive effect on villus height in all the intestinal segments, the increase was more pronounced in the HLS group compared to the NLS group. Our result is in agreement with a similar earlier study that recorded a significant increase in the villus height with *L. Plantarum* CGMCC supplemented a group of piglets and enhanced VH:CD^41^. The VH:CD in the ileum section of the intestinal gut was significantly increased in the treatment groups, which is an indicator of the increased superficial absorptive area with a thinner lamina propria in this vital part of the gut where most of the absorption of nutrients occurs.

Overall effects revealed that *L. salivarius* supplemented piglets (HLS and NLS) had no effects on ADG compared to the CON group. The average body weight of pigs in this experiment was 6.22 kg at weaning, which was in a similar range as previous reports^4,7,8,12,22,36^. This result is consistent with a previous study supplementing multi-strain probiotic including *L. acidophilus* that equally revealed a marginal difference in growth performance between the treatment group and the control group^12^. However, in a similar study, the addition of *L. casei* into the diet increased the ADG of piglets^7^. The insignificant ADG but significantly improved villus height may be explained by the physiological status and age of piglets, as we used the probiotics in suckling piglets with milk as the main feed source, however, most of the significant studies used probiotics for weaned piglets with a solid meal as the main feed source^7^. A greater villus height may result in a better performance after weaning when the diet changes from liquid milk to solid feed. The short period of the experiment can also be another reason for the insignificant results.

## Conclusion

In conclusion, based on the microbiota information, our study demonstrated that the population of beneficial bacteria such as *Lactobacillus* was significantly increased in the HLS-treated piglets. Moreover, the abundance of clostridia was decreased in the feces, which may emphasize the antimicrobial activity of HSL probiotic. These normal alterations in the gut microbiota at the suckling period decrease the susceptibility of weaned piglets to pathogenic infections at weaning time. The greater villus height of the duodenum, jejunum, and ileum can be considered as the indicators of the integrated intestine that may provide the potential for higher growth performance after weaning. This achievement may provide greater insight into the importance of intestinal microbiota manipulation during suckling, and future work focusing on the growth performance of weaned piglets seems necessary to confirm the improved growth potential in the suckling period.

## Materials and methods

### Animal care

This research was conducted according to the protocol approved by Kangwon National University institutional animal care and use committee (IACUC No.: KW-140509–1). All experiments were performed in accordance with relevant guidelines and regulations.

### Animals, experimental designs, and diets

The experiment was conducted at a commercial pig farm in Gangneung in the Republic of Korea. Standard farm management and husbandry practices were routinely carried out by the farm staff. Thirty cross-bred three-day-old piglets (1.54 ± 0.44 kg; Duroc × Yorkshire × Landrace) were randomly divided into three groups (n = 10, for each treatment). Cross fostering was done before starting the experiment. Each experiment litter was housed individually with the dam in individual stainless steel pens with reinforced plastic floors. Piglets had ad libitum access to sow milk and water. Sows were fed on a common corn-soybean meal-based diet. The treatments included the CON (basal diet; milk formula without probiotic), CON plus 20 ml/day of probiotic *L. salivarius*144 isolated from fast-growing pigs (HLS; 1 × 10^8^ cfu/ ml), CON plus 20 ml/day of probiotic *L. salivarius*160 isolated from normal weight pigs (NLS; 1 × 10^8^ cfu/ml). The basal dry milk formula was designed as a sow milk supplement. The mentioned *L. salivarius* were selected after passing the screening tests such as antimicrobial activity. The screened *L. salivarius* (LS144 and LS160) probiotics were acquired from Kangwon National University, Laboratory of Microbial Genomics and Big Data, and stored at 4 °C in individualized centrifugal tubes.

### *Lactobacillus salivarius* isolation and identification

*Lactobacillus salivarus* were isolated from the fecal samples of the fast-growing and normal weight of nine-week-old weaned pigs. The body weights of pigs (Landrace × Yorkshire × Duroc) were 15.35 ± 1.62 kg (mean ± SD) and 23.47 ± 2.11 kg (mean ± SD) for normal body weight and fast-growing pigs, respectively. Both groups fed with the same diet. To test the anti-pathogenic features, the isolated lactobacilli and *L. salivarius* KCTC 3600 as control were tested against *Salmonella* spp. as the most common pathogenic bacteria, which cause intestinal disease in swine^42^. After the screening process among the *L. salivarus* strains, two strains were isolated and identified as *L. salivarus* 160 (from normal weight pigs) and *L. salivarius* 144 (from fast-growing pigs). *L. salivarius* 144 and *L. salivarius* 160 species identification was based on species-primer sets targeting the genes^43^ and 16 s rRNA sequencing (*L. salivarius* 144, accession no. PRJNA669977; *L. salivarius* 160, accession no. PRJNA669979).

### Animal feeding and management

The fresh formula was provided two times daily (0800 h. and 1400 h.). The diets were reconstituted at 200 g dry milk formula diet in 800 ml of warm water at 40 °C. Then 10 ml (1 × 10^8^ cfu/ ml) of probiotic cultures (LS144 and LS160) was added to the HLS and NLS treatments and offered to the piglets by 10 nipples. Viable probiotic cultures as confirmed by the manufacturer, containers of the lyophilized probiotic were stored at 4 °C. Prior to the beginning of the experiment (day1) and at the end of the experiment (day 18), individual piglet weight was recorded for calculation of weight gain, and ADG. At the end of the experiment, piglets were euthanized by the approved anesthetic, and exsanguination and digesta and tissue samples were harvested immediately.

### Sample collection and analyses of intestinal digesta bacterial population

Digesta samples were obtained by stomach, duodenum, jejunum, ileum, and cecum puncturing for microbial population analysis as described by Hosseindoust et al.^4^. In short, one gram of digesta sample from each section of intestine including the duodenum, jejunum, ileum, and cecum was thoroughly mixed with 9 mL of sterile peptone PBS (0.1%). To determine the *Lactobacillus* spp. (using MRS agar + 0.200 g/l NaN3 + 0.500 g/l L‐cystine hydrochloride monohydrate, 48 h incubation at 37 °C; Difco Laboratories, Detroit), *Clostridium* spp. (TSC agar; 48 h incubation at 37 °C; Oxoid, Hampshire, UK) and coliforms (violet red bile agar, 24 h incubation at 37 °C; Merck Co., Ltd, Germany) were used. The bacterial concentration was calculated by the average of duplicate plates and expressed as (log, CFU/mL) before statistical analysis.

### Fecal bacterial population determination through

Using a NucleoSpin Soil kit (Macherey–Nagel, Duren, Germany), genomic DNA was extracted from 300 mg of each fecal sample as per the manufacturer’s recommendation then stored at − 72^°^ C awaiting analysis. The 16S ribosomal (rRNA) V4 region from the total extracted genomic DNA was amplified using Takara Ex-Taq DNA polymerase (Takara Bio, Shiga, Japan) and primer sets (forward: 5′-GGACTACHVGGGTWTCTAAT-3′ and reverse: 5′-GTGCCAGCMGCCGCGGTAA-3′). The amplification was performed in one cycle for 180 s (94 °C), following by 30 cycles for 45 s (94 °C), 60 s (55 °C), 90 s (72 °C), and one cycle for 10 min (72 °C). The separation and purification of amplicons were performed by using agarose gel electrophoresis, and QIAquick gel extraction kit (Qiagen, Valencia, CA, USA), respectively^23^. DNA library was sequenced on an Illumina MiSeq platform and paired-end sequence reads were generated which were then quality-trimmed and de-multiplexed using in-house Perl scripts. Filtered reads were processed and analyzed for microbial community diversity and richness indices using Quantitative Insights Into Microbial Ecology (QIIME 1.9.1)^23,44^. Each read was nominated as Operating Taxonomic Units (OTUs) when they showed a 97% sequencing identity with the Greengenes 13_8 database^45^. The OTUs were then normalized to 40,000 reads per sample by single rarefaction. Principal Coordinate Analysis (PCoA) was consequently drawn based on UniFrac distances as visualized with EMPeror Software^23^.

### Intestinal histomorphology

Mucosal and histological tissue samples were collected from the duodenum, jejunum, and ileum for intestinal histomorphology analysis and the remained samples were frozen in liquid nitrogen and stored at − 80 °C. The duodenal, jejunal, and ileal samples were cut approximately 5 cm, fixed in neutral buffered 10% formalin for 24 h, then transferred into a 70% ethanol solution and embedded in wax, sectioned, and stained with hematoxylin and eosin. Finally, the slices were each mounted on slides for analysis as previously described^46^. To measure the intestinal morphology, five well-defined villi and crypts from each section were identified. The villus height, measured from the villi tips up to villi-crypt junction were recorded along with the crypt depth, measured from the villi base at the lowest point of the crypt. The evaluation of intestinal sample slides was performed by using Vanox-S Microscope (Olympus Corporation, Lake Success, NY) then the images were analyzed using SPOT basic imaging software (Diagnostic Instruments, Sterling Heights, MI).

### Piglet performance

All the experimental animals were weighed individually on day one and the last day (day 18) of the experiment. This was used to calculate weight gain and ADG.

### Statistical analyses

Statistical analyses for parametric variables were done using the Mixed procedure of SAS package (version 9.4, SAS Inst., Cary, NC, USA) in a randomized complete block design for growth performance, culture-based intestinal digesta, and intestinal morphology. For nonparametric variables including taxonomic comparisons from 16S rDNA sequencing analysis, the significances among the groups were tested by nonparametric Kruskal–Wallis test. Bonferroni correction test was used as a correction for multiple comparisons. The alpha diversity indices were calculated by QIIME pipeline (alpha_diversity.py) through rarefaction with 10 iterations using OTUs. One-way analysis of variance (ANOVA) with Tukey’s post-hoc test and Kruskal–Wallis test were conducted using R software (version 4.0.2). Differences of P < 0.01 and or P < 0.05 were considered as statistically significant. PCoA was analyzed based on unweighted and weighted UniFrac distances, and the influences on the microbial community at different sampling stages were calculated using Adonis statistical tests using QIIME, with 999 permutations.

## Supplementary Information

Supplementary Information 1.

Supplementary Information 2.

## Competing of interests

The authors declare no compting of interests.
