# Author Correction: Effects of *Lactobacillus salivarius* isolated from feces of fast-growing pigs on intestinal microbiota and morphology of suckling piglets

**DOI:** 10.1038/s41598-021-88639-0

**Published:** 2021-04-21

**Authors:** Joseph Moturi, Kwang Yeol Kim, Abdolreza Hosseindoust, Jun Hyung Lee, Biao Xuan, Jongbin Park, Eun Bae Kim, Jin Soo Kim, Byung Jo Chae

**Affiliations:** 1 Department of Animal Industry Convergence, Kangwon National University, Chuncheon, 24341 Republic of Korea; 2 Kangwon National University, Chuncheon, 24341 Republic of Korea; 3 Poultry Research Institute, National Institute of Animal Science, Pyeongchang, 25342 Republic of Korea; 4 Department of Animal Resource Science, College of Animal Life Science, Kangwon National University, Chuncheon, 24341 Republic of Korea; 5 Department of Animal Biosciences, University of Guelph, Guelph, ON N1G 2W1 Canada; 6 Department of Applied Animal Science, College of Animal Life Science, Kangwon National University, Chuncheon, Kangwon‑do Republic of Korea

Correction to: *Scientific Reports* 10.1038/s41598-021-85630-7, published online 24 March 2021

The Acknowledgements section in this Article is incorrect.

“This study was supported by 2021 the RDA Fellowship Program of National Institute of Animal Science, Rural Development Administration, Republic of Korea.”

should read:

“This research was funded by Rural Development Administration, Korea (grant number: PJ0147962021).”

