# Supplementary material for: Effects of Lactobacillus salivarius isolated from feces of fast-growing pigs on intestinal microbiota and morphology of suckling piglets
Source: Sci Rep. 2021 Mar 24;11:6757. doi: 10.1038/s41598-021-85630-7 (PMC7990948; doi:10.1038/s41598-021-85630-7)
Supplement: Supplementary file 1 — Supplementary Information 1. [file 41598_2021_85630_MOESM1_ESM.pdf]

Effects of Lactobacillus salivarius isolated from feces of fast-growing pigs on intestinal microbiota and morphology of suckling piglets

Joseph Moturi, Kwang Yeol Kim, Abdolreza Hosseindoust, Min Ju Kim, Jun Hyung Lee, Biao Xuan, Jong Bin Park, Eun Bae Kim, and Byung Jo Chae
Correspondence Dr. B.J. Chae, Department of Animal Life Science, Kangwon National University, Chuncheon, 24341, Republic of Korea. Tel.: +82-33-250-8616; Fax: +82-33-244-4946, E-mail: bjchae@kangwon.ac.kr

	Group	G0001	G0001
	Individual	s001	s002
L2	k__Archaea;p__Euryarchaeota	0.01	0.01
L2	k__Bacteria;p__Acidobacteria	0	0
L2	k__Bacteria;p__Actinobacteria	0.72	0.2
L2	k__Bacteria;p__Bacteroidetes	53.8	53.9
L2	k__Bacteria;p__Chlamydiae	0	0
L2	k__Bacteria;p__Chloroflexi	0	0
L2	k__Bacteria;p__Cyanobacteria	0	0
L2	k__Bacteria;p__Deferribacteres	0.01	0
L2	k__Bacteria;p__Elusimicrobia	0	0
L2	k__Bacteria;p__Fibrobacteres	0	0
L2	k__Bacteria;p__Firmicutes	23.4	24
L2	k__Bacteria;p__Fusobacteria	13.1	18.9
L2	k__Bacteria;p__LD1	0	0
L2	k__Bacteria;p__Lentisphaerae	0	0.01
L2	k__Bacteria;p__Planctomycetes	0	0.01
L2	k__Bacteria;p__Proteobacteria	8.88	2.81
L2	k__Bacteria;p__SR1	0	0
L2	k__Bacteria;p__Spirochaetes	0.04	0.03
L2	k__Bacteria;p__Synergistetes	0	0.01
L2	k__Bacteria;p__TM6	0	0
L2	k__Bacteria;p__TM7	0	0
L2	k__Bacteria;p__Tenericutes	0.02	0.08
L2	k__Bacteria;p__Verrucomicrobia	0	0
L2	k__Bacteria;p__WPS-2	0	0
L2	k__Bacteria;p__[Thermi]	0	0
L6	k__Archaea;p__Euryarchaeota;c__Methanobacteria;o__Methanobacteriales;f__M	0	0
L6	k__Archaea;p__Euryarchaeota;c__Methanobacteria;o__Methanobacteriales;f__M	0.01	0.01
L6	k__Archaea;p__Euryarchaeota;c__Methanobacteria;o__Methanobacteriales;f__M	0	0
L6	k__Archaea;p__Euryarchaeota;c__Thermoplasmata;o__E2;f__[Methanomassiliicoc	0	0
L6	k__Bacteria;p__Acidobacteria;c__Acidobacteriia;o__Acidobacteriales;f__Acidobact	0	0
L6	k__Bacteria;p__Acidobacteria;c__Holophagae;o__Holophagales;f__Holophagaceae	0	0
L6	k__Bacteria;p__Actinobacteria;c__Actinobacteria;o__Actinomycetales;f__;g__	0	0
L6	k__Bacteria;p__Actinobacteria;c__Actinobacteria;o__Actinomycetales;f__Actinom	0	0
L6	k__Bacteria;p__Actinobacteria;c__Actinobacteria;o__Actinomycetales;f__Actinom	0	0
L6	k__Bacteria;p__Actinobacteria;c__Actinobacteria;o__Actinomycetales;f__Actinom	0	0
L6	k__Bacteria;p__Actinobacteria;c__Actinobacteria;o__Actinomycetales;f__Actinom	0	0
L6	k__Bacteria;p__Actinobacteria;c__Actinobacteria;o__Actinomycetales;f__Actinom	0	0
L6	k__Bacteria;p__Actinobacteria;c__Actinobacteria;o__Actinomycetales;f__Actinom	0	0
L6	k__Bacteria;p__Actinobacteria;c__Actinobacteria;o__Actinomycetales;f__Beutenb	0	0
L6	k__Bacteria;p__Actinobacteria;c__Actinobacteria;o__Actinomycetales;f__Brevibac	0	0
L6	k__Bacteria;p__Actinobacteria;c__Actinobacteria;o__Actinomycetales;f__Coryneb	0.14	0.04

L6	k__Bacteria;p__Actinobacteria;c__Actinobacteria;o__Actinomycetales;f__Dermaba	0	0
L6	k__Bacteria;p__Actinobacteria;c__Actinobacteria;o__Actinomycetales;f__Dermaba	0	0
L6	k__Bacteria;p__Actinobacteria;c__Actinobacteria;o__Actinomycetales;f__Dermaba	0	0
L6	k__Bacteria;p__Actinobacteria;c__Actinobacteria;o__Actinomycetales;f__Dermacc	0	0
L6	k__Bacteria;p__Actinobacteria;c__Actinobacteria;o__Actinomycetales;f__Dermatc	0	0
L6	k__Bacteria;p__Actinobacteria;c__Actinobacteria;o__Actinomycetales;f__Dietziace	0	0
L6	k__Bacteria;p__Actinobacteria;c__Actinobacteria;o__Actinomycetales;f__Dietziace	0	0
L6	k__Bacteria;p__Actinobacteria;c__Actinobacteria;o__Actinomycetales;f__Geodern	0	0
L6	k__Bacteria;p__Actinobacteria;c__Actinobacteria;o__Actinomycetales;f__Intraspo	0	0
L6	k__Bacteria;p__Actinobacteria;c__Actinobacteria;o__Actinomycetales;f__Intraspo	0	0
L6	k__Bacteria;p__Actinobacteria;c__Actinobacteria;o__Actinomycetales;f__Intraspo	0	0
L6	k__Bacteria;p__Actinobacteria;c__Actinobacteria;o__Actinomycetales;f__Intraspo	0	0
L6	k__Bacteria;p__Actinobacteria;c__Actinobacteria;o__Actinomycetales;f__Microba	0	0
L6	k__Bacteria;p__Actinobacteria;c__Actinobacteria;o__Actinomycetales;f__Microba	0	0
L6	k__Bacteria;p__Actinobacteria;c__Actinobacteria;o__Actinomycetales;f__Microba	0	0
L6	k__Bacteria;p__Actinobacteria;c__Actinobacteria;o__Actinomycetales;f__Microba	0	0
L6	k__Bacteria;p__Actinobacteria;c__Actinobacteria;o__Actinomycetales;f__Microba	0	0
L6	k__Bacteria;p__Actinobacteria;c__Actinobacteria;o__Actinomycetales;f__Microba	0	0
L6	k__Bacteria;p__Actinobacteria;c__Actinobacteria;o__Actinomycetales;f__Microba	0	0
L6	k__Bacteria;p__Actinobacteria;c__Actinobacteria;o__Actinomycetales;f__Microco	0.26	0.05
L6	k__Bacteria;p__Actinobacteria;c__Actinobacteria;o__Actinomycetales;f__Microco	0	0
L6	k__Bacteria;p__Actinobacteria;c__Actinobacteria;o__Actinomycetales;f__Microco	0	0
L6	k__Bacteria;p__Actinobacteria;c__Actinobacteria;o__Actinomycetales;f__Microco	0	0
L6	k__Bacteria;p__Actinobacteria;c__Actinobacteria;o__Actinomycetales;f__Microco	0	0
L6	k__Bacteria;p__Actinobacteria;c__Actinobacteria;o__Actinomycetales;f__Microco	0.01	0
L6	k__Bacteria;p__Actinobacteria;c__Actinobacteria;o__Actinomycetales;f__Nocardia	0	0
L6	k__Bacteria;p__Actinobacteria;c__Actinobacteria;o__Actinomycetales;f__Nocardic	0	0
L6	k__Bacteria;p__Actinobacteria;c__Actinobacteria;o__Actinomycetales;f__Nocardic	0	0
L6	k__Bacteria;p__Actinobacteria;c__Actinobacteria;o__Actinomycetales;f__Nocardic	0	0
L6	k__Bacteria;p__Actinobacteria;c__Actinobacteria;o__Actinomycetales;f__Nocardic	0	0
L6	k__Bacteria;p__Actinobacteria;c__Actinobacteria;o__Actinomycetales;f__Promicro	0	0
L6	k__Bacteria;p__Actinobacteria;c__Actinobacteria;o__Actinomycetales;f__Propioni	0	0
L6	k__Bacteria;p__Actinobacteria;c__Actinobacteria;o__Actinomycetales;f__Propioni	0	0
L6	k__Bacteria;p__Actinobacteria;c__Actinobacteria;o__Actinomycetales;f__Pseudon	0	0
L6	k__Bacteria;p__Actinobacteria;c__Actinobacteria;o__Actinomycetales;f__Pseudon	0	0
L6	k__Bacteria;p__Actinobacteria;c__Actinobacteria;o__Actinomycetales;f__Yaniellac	0	0
L6	k__Bacteria;p__Actinobacteria;c__Actinobacteria;o__Actinomycetales;f__Yaniellac	0	0
L6	k__Bacteria;p__Actinobacteria;c__Actinobacteria;o__Bifidobacteriales;f__Bifidoba	0	0
L6	k__Bacteria;p__Actinobacteria;c__Actinobacteria;o__Bifidobacteriales;f__Bifidoba	0.2	0.01
L6	k__Bacteria;p__Actinobacteria;c__Actinobacteria;o__Bifidobacteriales;f__Bifidoba	0	0
L6	k__Bacteria;p__Actinobacteria;c__Coriobacteriia;o__Coriobacteriales;f__Coriobact	0.08	0.01
L6	k__Bacteria;p__Actinobacteria;c__Coriobacteriia;o__Coriobacteriales;f__Coriobact	0	0
L6	k__Bacteria;p__Actinobacteria;c__Coriobacteriia;o__Coriobacteriales;f__Coriobact	0	0
L6	k__Bacteria;p__Actinobacteria;c__Coriobacteriia;o__Coriobacteriales;f__Coriobact	0.01	0.03
L6	k__Bacteria;p__Actinobacteria;c__Coriobacteriia;o__Coriobacteriales;f__Coriobact	0.01	0.02
L6	k__Bacteria;p__Actinobacteria;c__Coriobacteriia;o__Coriobacteriales;f__Coriobact	0	0
L6	k__Bacteria;p__Actinobacteria;c__Coriobacteriia;o__Coriobacteriales;f__Coriobact	0	0
L6	k__Bacteria;p__Actinobacteria;c__Rubrobacteria;o__Rubrobacterales;f__Rubrobac	0	0.01
L6	k__Bacteria;p__Bacteroidetes;c__Bacteroidia;o__Bacteroidales;f__;g__	0.22	0.4
L6	k__Bacteria;p__Bacteroidetes;c__Bacteroidia;o__Bacteroidales;f__BS11;g__	0	0

L6	k__Bacteria;p__Bacteroidetes;c__Bacteroidia;o__Bacteroidales;f__Bacteroidaceae	0	0
L6	k__Bacteria;p__Bacteroidetes;c__Bacteroidia;o__Bacteroidales;f__Bacteroidaceae	0	0
L6	k__Bacteria;p__Bacteroidetes;c__Bacteroidia;o__Bacteroidales;f__Bacteroidaceae	0	0
L6	k__Bacteria;p__Bacteroidetes;c__Bacteroidia;o__Bacteroidales;f__Bacteroidaceae	15.3	34.7
L6	k__Bacteria;p__Bacteroidetes;c__Bacteroidia;o__Bacteroidales;f__Porphyromona	0.02	0
L6	k__Bacteria;p__Bacteroidetes;c__Bacteroidia;o__Bacteroidales;f__Porphyromona	0	0
L6	k__Bacteria;p__Bacteroidetes;c__Bacteroidia;o__Bacteroidales;f__Porphyromona	0	0
L6	k__Bacteria;p__Bacteroidetes;c__Bacteroidia;o__Bacteroidales;f__Porphyromona	0.63	0.67
L6	k__Bacteria;p__Bacteroidetes;c__Bacteroidia;o__Bacteroidales;f__Porphyromona	0	0
L6	k__Bacteria;p__Bacteroidetes;c__Bacteroidia;o__Bacteroidales;f__Prevotellaceae;	0	0
L6	k__Bacteria;p__Bacteroidetes;c__Bacteroidia;o__Bacteroidales;f__Prevotellaceae;	28.6	1.57
L6	k__Bacteria;p__Bacteroidetes;c__Bacteroidia;o__Bacteroidales;f__RF16;g__	0	0
L6	k__Bacteria;p__Bacteroidetes;c__Bacteroidia;o__Bacteroidales;f__Rikenellaceae;g	0.04	0.12
L6	k__Bacteria;p__Bacteroidetes;c__Bacteroidia;o__Bacteroidales;f__Rikenellaceae;g	0	0
L6	k__Bacteria;p__Bacteroidetes;c__Bacteroidia;o__Bacteroidales;f__S24-7;g__	3.04	8.36
L6	k__Bacteria;p__Bacteroidetes;c__Bacteroidia;o__Bacteroidales;f__[Barnesiellacea	0	0
L6	k__Bacteria;p__Bacteroidetes;c__Bacteroidia;o__Bacteroidales;f__[Odoribacterac	5.33	6.64
L6	k__Bacteria;p__Bacteroidetes;c__Bacteroidia;o__Bacteroidales;f__[Odoribacterac	0.01	0
L6	k__Bacteria;p__Bacteroidetes;c__Bacteroidia;o__Bacteroidales;f__[Paraprevotella	0.01	0.11
L6	k__Bacteria;p__Bacteroidetes;c__Bacteroidia;o__Bacteroidales;f__[Paraprevotella	0.19	0.03
L6	k__Bacteria;p__Bacteroidetes;c__Bacteroidia;o__Bacteroidales;f__[Paraprevotella	0	0
L6	k__Bacteria;p__Bacteroidetes;c__Bacteroidia;o__Bacteroidales;f__[Paraprevotella	0.01	0.01
L6	k__Bacteria;p__Bacteroidetes;c__Bacteroidia;o__Bacteroidales;f__[Paraprevotella	0.11	0.77
L6	k__Bacteria;p__Bacteroidetes;c__Bacteroidia;o__Bacteroidales;f__p-2534-18B5;g_	0.3	0.49
L6	k__Bacteria;p__Bacteroidetes;c__Cytophagia;o__Cytophagales;f__Cytophagaceae;	0	0
L6	k__Bacteria;p__Bacteroidetes;c__Cytophagia;o__Cytophagales;f__[Amoebophilace	0	0
L6	k__Bacteria;p__Bacteroidetes;c__Flavobacteriia;o__Flavobacteriales;f__Flavobacte	0	0
L6	k__Bacteria;p__Bacteroidetes;c__Flavobacteriia;o__Flavobacteriales;f__Flavobacte	0	0
L6	k__Bacteria;p__Bacteroidetes;c__Flavobacteriia;o__Flavobacteriales;f__[Weeksell	0.01	0
L6	k__Bacteria;p__Bacteroidetes;c__Flavobacteriia;o__Flavobacteriales;f__[Weeksell	0	0
L6	k__Bacteria;p__Bacteroidetes;c__Flavobacteriia;o__Flavobacteriales;f__[Weeksell	0	0
L6	k__Bacteria;p__Bacteroidetes;c__Flavobacteriia;o__Flavobacteriales;f__[Weeksell	0	0
L6	k__Bacteria;p__Bacteroidetes;c__Flavobacteriia;o__Flavobacteriales;f__[Weeksell	0	0
L6	k__Bacteria;p__Bacteroidetes;c__Sphingobacteriia;o__Sphingobacteriales;f__Sphi	0	0
L6	k__Bacteria;p__Bacteroidetes;c__Sphingobacteriia;o__Sphingobacteriales;f__Sphi	0	0
L6	k__Bacteria;p__Bacteroidetes;c__[Saprospirae];o__[Saprospirales];f__Chitinophag	0	0
L6	k__Bacteria;p__Bacteroidetes;c__[Saprospirae];o__[Saprospirales];f__Chitinophag	0	0.01
L6	k__Bacteria;p__Bacteroidetes;c__[Saprospirae];o__[Saprospirales];f__Chitinophag	0	0
L6	k__Bacteria;p__Chlamydiae;c__Chlamydiia;o__Chlamydiales;f__Chlamydiaceae;g_	0	0
L6	k__Bacteria;p__Chloroflexi;c__Anaerolineae;o__Anaerolineales;f__Anaerolinaceae	0	0
L6	k__Bacteria;p__Chloroflexi;c__Anaerolineae;o__Anaerolineales;f__Anaerolinaceae	0	0
L6	k__Bacteria;p__Chloroflexi;c__Thermomicrobia;o__JG30-KF-CM45;f__;g__	0	0
L6	k__Bacteria;p__Cyanobacteria;c__4C0d-2;o__YS2;f__;g__	0	0
L6	k__Bacteria;p__Cyanobacteria;c__Chloroplast;o__Streptophyta;f__;g__	0	0
L6	k__Bacteria;p__Deferribacteres;c__Deferribacteres;o__Deferribacterales;f__Defer	0.01	0
L6	k__Bacteria;p__Elusimicrobia;c__Elusimicrobia;o__Elusimicrobiales;f__Elusimicrob	0	0
L6	k__Bacteria;p__Elusimicrobia;c__Elusimicrobia;o__Elusimicrobiales;f__Elusimicrob	0	0
L6	k__Bacteria;p__Elusimicrobia;c__Endomicrobia;o__;f__;g__	0	0
L6	k__Bacteria;p__Fibrobacteres;c__Fibrobacteria;o__Fibrobacterales;f__Fibrobacter	0	0
L6	k__Bacteria;p__Firmicutes;c__Bacilli;o__Bacillales;f__;g__	0	0

L6	k__Bacteria;p__Firmicutes;c__Bacilli;o__Bacillales;f__Bacillaceae;g__	0	0
L6	k__Bacteria;p__Firmicutes;c__Bacilli;o__Bacillales;f__Bacillaceae;g__Anaerobacillu	0	0
L6	k__Bacteria;p__Firmicutes;c__Bacilli;o__Bacillales;f__Bacillaceae;g__Anoxybacillus	0	0
L6	k__Bacteria;p__Firmicutes;c__Bacilli;o__Bacillales;f__Bacillaceae;g__Bacillus	0	0
L6	k__Bacteria;p__Firmicutes;c__Bacilli;o__Bacillales;f__Bacillaceae;g__Salimicrobiun	0	0
L6	k__Bacteria;p__Firmicutes;c__Bacilli;o__Bacillales;f__Planococcaceae;g__	0	0
L6	k__Bacteria;p__Firmicutes;c__Bacilli;o__Bacillales;f__Planococcaceae;g__Lysinibac	0	0
L6	k__Bacteria;p__Firmicutes;c__Bacilli;o__Bacillales;f__Planococcaceae;g__Planomic	0	0
L6	k__Bacteria;p__Firmicutes;c__Bacilli;o__Bacillales;f__Planococcaceae;g__Rummeli	0	0
L6	k__Bacteria;p__Firmicutes;c__Bacilli;o__Bacillales;f__Planococcaceae;g__Solibacill	0	0
L6	k__Bacteria;p__Firmicutes;c__Bacilli;o__Bacillales;f__Planococcaceae;g__Sporosar	0	0
L6	k__Bacteria;p__Firmicutes;c__Bacilli;o__Bacillales;f__Staphylococcaceae;g__	0	0
L6	k__Bacteria;p__Firmicutes;c__Bacilli;o__Bacillales;f__Staphylococcaceae;g__Jeotg	0	0
L6	k__Bacteria;p__Firmicutes;c__Bacilli;o__Bacillales;f__Staphylococcaceae;g__Macr	0	0
L6	k__Bacteria;p__Firmicutes;c__Bacilli;o__Bacillales;f__Staphylococcaceae;g__Salini	0	0
L6	k__Bacteria;p__Firmicutes;c__Bacilli;o__Bacillales;f__Staphylococcaceae;g__Staph	0.02	0.01
L6	k__Bacteria;p__Firmicutes;c__Bacilli;o__Bacillales;f__[Exiguobacteraceae];g__Exig	0	0
L6	k__Bacteria;p__Firmicutes;c__Bacilli;o__Gemellales;f__;g__	0	0
L6	k__Bacteria;p__Firmicutes;c__Bacilli;o__Gemellales;f__Gemellaceae;g__	0	0
L6	k__Bacteria;p__Firmicutes;c__Bacilli;o__Gemellales;f__Gemellaceae;g__Gemella	0	0
L6	k__Bacteria;p__Firmicutes;c__Bacilli;o__Lactobacillales;f__;g__	0	0
L6	k__Bacteria;p__Firmicutes;c__Bacilli;o__Lactobacillales;f__Aerococcaceae;g__	0	0
L6	k__Bacteria;p__Firmicutes;c__Bacilli;o__Lactobacillales;f__Aerococcaceae;g__Aer	0	0
L6	k__Bacteria;p__Firmicutes;c__Bacilli;o__Lactobacillales;f__Aerococcaceae;g__Alka	0	0
L6	k__Bacteria;p__Firmicutes;c__Bacilli;o__Lactobacillales;f__Aerococcaceae;g__Alloi	0	0
L6	k__Bacteria;p__Firmicutes;c__Bacilli;o__Lactobacillales;f__Aerococcaceae;g__Fack	0	0.01
L6	k__Bacteria;p__Firmicutes;c__Bacilli;o__Lactobacillales;f__Carnobacteriaceae;g__	0	0
L6	k__Bacteria;p__Firmicutes;c__Bacilli;o__Lactobacillales;f__Carnobacteriaceae;g__	0	0
L6	k__Bacteria;p__Firmicutes;c__Bacilli;o__Lactobacillales;f__Carnobacteriaceae;g__	0	0
L6	k__Bacteria;p__Firmicutes;c__Bacilli;o__Lactobacillales;f__Carnobacteriaceae;g__	0	0
L6	k__Bacteria;p__Firmicutes;c__Bacilli;o__Lactobacillales;f__Carnobacteriaceae;g__	0	0
L6	k__Bacteria;p__Firmicutes;c__Bacilli;o__Lactobacillales;f__Enterococcaceae;g__	0	0
L6	k__Bacteria;p__Firmicutes;c__Bacilli;o__Lactobacillales;f__Enterococcaceae;g__En	0.02	0.03
L6	k__Bacteria;p__Firmicutes;c__Bacilli;o__Lactobacillales;f__Enterococcaceae;g__Va	0	0
L6	k__Bacteria;p__Firmicutes;c__Bacilli;o__Lactobacillales;f__Lactobacillaceae;g__	0	0
L6	k__Bacteria;p__Firmicutes;c__Bacilli;o__Lactobacillales;f__Lactobacillaceae;g__Lac	2.2	2.07
L6	k__Bacteria;p__Firmicutes;c__Bacilli;o__Lactobacillales;f__Lactobacillaceae;g__Pe	0	0
L6	k__Bacteria;p__Firmicutes;c__Bacilli;o__Lactobacillales;f__Leuconostocaceae;g__	0	0
L6	k__Bacteria;p__Firmicutes;c__Bacilli;o__Lactobacillales;f__Leuconostocaceae;g__L	0	0
L6	k__Bacteria;p__Firmicutes;c__Bacilli;o__Lactobacillales;f__Leuconostocaceae;g__V	0	0
L6	k__Bacteria;p__Firmicutes;c__Bacilli;o__Lactobacillales;f__Streptococcaceae;g__	0	0
L6	k__Bacteria;p__Firmicutes;c__Bacilli;o__Lactobacillales;f__Streptococcaceae;g__La	0	0
L6	k__Bacteria;p__Firmicutes;c__Bacilli;o__Lactobacillales;f__Streptococcaceae;g__St	1.71	1.1
L6	k__Bacteria;p__Firmicutes;c__Bacilli;o__Turicibacterales;f__Turicibacteraceae;g__	0.02	0.02
L6	k__Bacteria;p__Firmicutes;c__Clostridia;o__;f__;g__	0	0
L6	k__Bacteria;p__Firmicutes;c__Clostridia;o__Clostridiales;f__;g__	1.96	5.83
L6	k__Bacteria;p__Firmicutes;c__Clostridia;o__Clostridiales;f__Christensenellaceae;g_	0.11	0.16
L6	k__Bacteria;p__Firmicutes;c__Clostridia;o__Clostridiales;f__Christensenellaceae;g_	0	0
L6	k__Bacteria;p__Firmicutes;c__Clostridia;o__Clostridiales;f__Clostridiaceae;g__	0.53	0.28
L6	k__Bacteria;p__Firmicutes;c__Clostridia;o__Clostridiales;f__Clostridiaceae;g__02d	0	0

L6	k__Bacteria;p__Firmicutes;c__Clostridia;o__Clostridiales;f__Clostridiaceae;g__Can	0	0
L6	k__Bacteria;p__Firmicutes;c__Clostridia;o__Clostridiales;f__Clostridiaceae;g__Clos	4.43	2.31
L6	k__Bacteria;p__Firmicutes;c__Clostridia;o__Clostridiales;f__Clostridiaceae;g__Prot	0	0
L6	k__Bacteria;p__Firmicutes;c__Clostridia;o__Clostridiales;f__Clostridiaceae;g__SMB	0.14	0.07
L6	k__Bacteria;p__Firmicutes;c__Clostridia;o__Clostridiales;f__Clostridiaceae;g__Sarc	0	0
L6	k__Bacteria;p__Firmicutes;c__Clostridia;o__Clostridiales;f__Dehalobacteriaceae;g_	0	0
L6	k__Bacteria;p__Firmicutes;c__Clostridia;o__Clostridiales;f__Dehalobacteriaceae;g_	0	0
L6	k__Bacteria;p__Firmicutes;c__Clostridia;o__Clostridiales;f__EtOH8;g__	0	0
L6	k__Bacteria;p__Firmicutes;c__Clostridia;o__Clostridiales;f__Eubacteriaceae;g__An	0	0
L6	k__Bacteria;p__Firmicutes;c__Clostridia;o__Clostridiales;f__Eubacteriaceae;g__Pse	0.01	0
L6	k__Bacteria;p__Firmicutes;c__Clostridia;o__Clostridiales;f__Lachnospiraceae;g__	1.15	1.16
L6	k__Bacteria;p__Firmicutes;c__Clostridia;o__Clostridiales;f__Lachnospiraceae;g__A	0	0
L6	k__Bacteria;p__Firmicutes;c__Clostridia;o__Clostridiales;f__Lachnospiraceae;g__B	0.05	0.04
L6	k__Bacteria;p__Firmicutes;c__Clostridia;o__Clostridiales;f__Lachnospiraceae;g__B	0	0
L6	k__Bacteria;p__Firmicutes;c__Clostridia;o__Clostridiales;f__Lachnospiraceae;g__C	0	0
L6	k__Bacteria;p__Firmicutes;c__Clostridia;o__Clostridiales;f__Lachnospiraceae;g__C	0.07	0.04
L6	k__Bacteria;p__Firmicutes;c__Clostridia;o__Clostridiales;f__Lachnospiraceae;g__D	0.26	0.76
L6	k__Bacteria;p__Firmicutes;c__Clostridia;o__Clostridiales;f__Lachnospiraceae;g__E	0	0
L6	k__Bacteria;p__Firmicutes;c__Clostridia;o__Clostridiales;f__Lachnospiraceae;g__La	0	0
L6	k__Bacteria;p__Firmicutes;c__Clostridia;o__Clostridiales;f__Lachnospiraceae;g__La	0	0
L6	k__Bacteria;p__Firmicutes;c__Clostridia;o__Clostridiales;f__Lachnospiraceae;g__P	0	0
L6	k__Bacteria;p__Firmicutes;c__Clostridia;o__Clostridiales;f__Lachnospiraceae;g__R	0.01	0.07
L6	k__Bacteria;p__Firmicutes;c__Clostridia;o__Clostridiales;f__Lachnospiraceae;g__Sl	0.02	0.01
L6	k__Bacteria;p__Firmicutes;c__Clostridia;o__Clostridiales;f__Lachnospiraceae;g__[I	0.3	0.81
L6	k__Bacteria;p__Firmicutes;c__Clostridia;o__Clostridiales;f__Peptococcaceae;g__	0	0
L6	k__Bacteria;p__Firmicutes;c__Clostridia;o__Clostridiales;f__Peptococcaceae;g__De	0	0
L6	k__Bacteria;p__Firmicutes;c__Clostridia;o__Clostridiales;f__Peptococcaceae;g__Pe	0.01	0.01
L6	k__Bacteria;p__Firmicutes;c__Clostridia;o__Clostridiales;f__Peptococcaceae;g__rc	0	0
L6	k__Bacteria;p__Firmicutes;c__Clostridia;o__Clostridiales;f__Peptostreptococcacea	0.05	0.01
L6	k__Bacteria;p__Firmicutes;c__Clostridia;o__Clostridiales;f__Peptostreptococcacea	0	0
L6	k__Bacteria;p__Firmicutes;c__Clostridia;o__Clostridiales;f__Peptostreptococcacea	0.13	0
L6	k__Bacteria;p__Firmicutes;c__Clostridia;o__Clostridiales;f__Peptostreptococcacea	2.06	0.22
L6	k__Bacteria;p__Firmicutes;c__Clostridia;o__Clostridiales;f__Ruminococcaceae;g__	0.62	2.71
L6	k__Bacteria;p__Firmicutes;c__Clostridia;o__Clostridiales;f__Ruminococcaceae;g__	0	0
L6	k__Bacteria;p__Firmicutes;c__Clostridia;o__Clostridiales;f__Ruminococcaceae;g__	0	0.02
L6	k__Bacteria;p__Firmicutes;c__Clostridia;o__Clostridiales;f__Ruminococcaceae;g__	0.48	0.49
L6	k__Bacteria;p__Firmicutes;c__Clostridia;o__Clostridiales;f__Ruminococcaceae;g__	0.02	0
L6	k__Bacteria;p__Firmicutes;c__Clostridia;o__Clostridiales;f__Ruminococcaceae;g__	0.02	0.02
L6	k__Bacteria;p__Firmicutes;c__Clostridia;o__Clostridiales;f__Ruminococcaceae;g__	0.78	2.21
L6	k__Bacteria;p__Firmicutes;c__Clostridia;o__Clostridiales;f__Ruminococcaceae;g__	0.08	1.32
L6	k__Bacteria;p__Firmicutes;c__Clostridia;o__Clostridiales;f__Veillonellaceae;g__	0.02	0.03
L6	k__Bacteria;p__Firmicutes;c__Clostridia;o__Clostridiales;f__Veillonellaceae;g__Aci	0.02	0.01
L6	k__Bacteria;p__Firmicutes;c__Clostridia;o__Clostridiales;f__Veillonellaceae;g__An	0	0
L6	k__Bacteria;p__Firmicutes;c__Clostridia;o__Clostridiales;f__Veillonellaceae;g__Dia	1.17	0.01
L6	k__Bacteria;p__Firmicutes;c__Clostridia;o__Clostridiales;f__Veillonellaceae;g__Me	0.37	0.09
L6	k__Bacteria;p__Firmicutes;c__Clostridia;o__Clostridiales;f__Veillonellaceae;g__Mi	0.02	0.02
L6	k__Bacteria;p__Firmicutes;c__Clostridia;o__Clostridiales;f__Veillonellaceae;g__Ph	1.13	1.51
L6	k__Bacteria;p__Firmicutes;c__Clostridia;o__Clostridiales;f__Veillonellaceae;g__Sel	0	0
L6	k__Bacteria;p__Firmicutes;c__Clostridia;o__Clostridiales;f__Veillonellaceae;g__Sp	0	0
L6	k__Bacteria;p__Firmicutes;c__Clostridia;o__Clostridiales;f__Veillonellaceae;g__Su	0	0

L6	k__Bacteria;p__Firmicutes;c__Clostridia;o__Clostridiales;f__Veillonellaceae;g__Suc	0	0
L6	k__Bacteria;p__Firmicutes;c__Clostridia;o__Clostridiales;f__Veillonellaceae;g__Vei	2.25	0.05
L6	k__Bacteria;p__Firmicutes;c__Clostridia;o__Clostridiales;f__[Acidaminobacteracea	0	0
L6	k__Bacteria;p__Firmicutes;c__Clostridia;o__Clostridiales;f__[Mogibacteriaceae];g_	0.1	0.03
L6	k__Bacteria;p__Firmicutes;c__Clostridia;o__Clostridiales;f__[Mogibacteriaceae];g_	0	0
L6	k__Bacteria;p__Firmicutes;c__Clostridia;o__Clostridiales;f__[Mogibacteriaceae];g_	0	0
L6	k__Bacteria;p__Firmicutes;c__Clostridia;o__Clostridiales;f__[Tissierellaceae];g__	0	0
L6	k__Bacteria;p__Firmicutes;c__Clostridia;o__Clostridiales;f__[Tissierellaceae];g__1-	0	0
L6	k__Bacteria;p__Firmicutes;c__Clostridia;o__Clostridiales;f__[Tissierellaceae];g__Ar	0.34	0.01
L6	k__Bacteria;p__Firmicutes;c__Clostridia;o__Clostridiales;f__[Tissierellaceae];g__Fi	0.02	0.01
L6	k__Bacteria;p__Firmicutes;c__Clostridia;o__Clostridiales;f__[Tissierellaceae];g__G\	0	0
L6	k__Bacteria;p__Firmicutes;c__Clostridia;o__Clostridiales;f__[Tissierellaceae];g__Ga	0	0.02
L6	k__Bacteria;p__Firmicutes;c__Clostridia;o__Clostridiales;f__[Tissierellaceae];g__He	0	0
L6	k__Bacteria;p__Firmicutes;c__Clostridia;o__Clostridiales;f__[Tissierellaceae];g__Pa	0	0
L6	k__Bacteria;p__Firmicutes;c__Clostridia;o__Clostridiales;f__[Tissierellaceae];g__Pe	0.11	0
L6	k__Bacteria;p__Firmicutes;c__Clostridia;o__Clostridiales;f__[Tissierellaceae];g__Ti:	0	0
L6	k__Bacteria;p__Firmicutes;c__Clostridia;o__Clostridiales;f__[Tissierellaceae];g__W	0	0
L6	k__Bacteria;p__Firmicutes;c__Clostridia;o__Clostridiales;f__[Tissierellaceae];g__ph	0	0
L6	k__Bacteria;p__Firmicutes;c__Clostridia;o__SHA-98;f__;g__	0	0
L6	k__Bacteria;p__Firmicutes;c__Erysipelotrichi;o__Erysipelotrichales;f__Erysipelotric	0.01	0.01
L6	k__Bacteria;p__Firmicutes;c__Erysipelotrichi;o__Erysipelotrichales;f__Erysipelotric	0.29	0.02
L6	k__Bacteria;p__Firmicutes;c__Erysipelotrichi;o__Erysipelotrichales;f__Erysipelotric	0	0
L6	k__Bacteria;p__Firmicutes;c__Erysipelotrichi;o__Erysipelotrichales;f__Erysipelotric	0	0
L6	k__Bacteria;p__Firmicutes;c__Erysipelotrichi;o__Erysipelotrichales;f__Erysipelotric	0	0
L6	k__Bacteria;p__Firmicutes;c__Erysipelotrichi;o__Erysipelotrichales;f__Erysipelotric	0	0
L6	k__Bacteria;p__Firmicutes;c__Erysipelotrichi;o__Erysipelotrichales;f__Erysipelotric	0	0
L6	k__Bacteria;p__Firmicutes;c__Erysipelotrichi;o__Erysipelotrichales;f__Erysipelotric	0	0
L6	k__Bacteria;p__Firmicutes;c__Erysipelotrichi;o__Erysipelotrichales;f__Erysipelotric	0	0
L6	k__Bacteria;p__Firmicutes;c__Erysipelotrichi;o__Erysipelotrichales;f__Erysipelotric	0.02	0.02
L6	k__Bacteria;p__Firmicutes;c__Erysipelotrichi;o__Erysipelotrichales;f__Erysipelotric	0.15	0.11
L6	k__Bacteria;p__Firmicutes;c__Erysipelotrichi;o__Erysipelotrichales;f__Erysipelotric	0.07	0.25
L6	k__Bacteria;p__Fusobacteria;c__Fusobacteriia;o__Fusobacteriales;f__Fusobacteria	0.04	0.12
L6	k__Bacteria;p__Fusobacteria;c__Fusobacteriia;o__Fusobacteriales;f__Fusobacteria	0	0
L6	k__Bacteria;p__Fusobacteria;c__Fusobacteriia;o__Fusobacteriales;f__Fusobacteria	13	18.8
L6	k__Bacteria;p__Fusobacteria;c__Fusobacteriia;o__Fusobacteriales;f__Leptotrichiac	0	0
L6	k__Bacteria;p__LD1;c__;o__;f__;g__	0	0
L6	k__Bacteria;p__Lentisphaerae;c__[Lentisphaeria];o__Victivallales;f__Victivallaceae	0	0
L6	k__Bacteria;p__Lentisphaerae;c__[Lentisphaeria];o__Victivallales;f__Victivallaceae	0	0
L6	k__Bacteria;p__Lentisphaerae;c__[Lentisphaeria];o__Z20;f__R4-45B;g__	0	0
L6	k__Bacteria;p__Planctomycetes;c__Planctomycetia;o__Gemmatales;f__Isosphaera	0	0
L6	k__Bacteria;p__Planctomycetes;c__Planctomycetia;o__Pirellulales;f__Pirellulaceae	0	0.01
L6	k__Bacteria;p__Proteobacteria;c__Alphaproteobacteria;o__;f__;g__	0	0
L6	k__Bacteria;p__Proteobacteria;c__Alphaproteobacteria;o__BD7-3;f__;g__	0	0
L6	k__Bacteria;p__Proteobacteria;c__Alphaproteobacteria;o__Caulobacterales;f__Ca	0	0
L6	k__Bacteria;p__Proteobacteria;c__Alphaproteobacteria;o__Caulobacterales;f__Ca	0	0
L6	k__Bacteria;p__Proteobacteria;c__Alphaproteobacteria;o__Caulobacterales;f__Ca	0	0
L6	k__Bacteria;p__Proteobacteria;c__Alphaproteobacteria;o__Caulobacterales;f__Ca	0	0
L6	k__Bacteria;p__Proteobacteria;c__Alphaproteobacteria;o__Caulobacterales;f__Ca	0	0
L6	k__Bacteria;p__Proteobacteria;c__Alphaproteobacteria;o__RF32;f__;g__	0	0
L6	k__Bacteria;p__Proteobacteria;c__Alphaproteobacteria;o__Rhizobiales;f__;g__	0	0

L6	k__Bacteria;p__Proteobacteria;c__Alphaproteobacteria;o__Rhizobiales;f__Auranti	0	0
L6	k__Bacteria;p__Proteobacteria;c__Alphaproteobacteria;o__Rhizobiales;f__Auranti	0	0
L6	k__Bacteria;p__Proteobacteria;c__Alphaproteobacteria;o__Rhizobiales;f__Bartone	0	0
L6	k__Bacteria;p__Proteobacteria;c__Alphaproteobacteria;o__Rhizobiales;f__Beijerin	0	0
L6	k__Bacteria;p__Proteobacteria;c__Alphaproteobacteria;o__Rhizobiales;f__Bradyrh	0	0.01
L6	k__Bacteria;p__Proteobacteria;c__Alphaproteobacteria;o__Rhizobiales;f__Bradyrh	0	0
L6	k__Bacteria;p__Proteobacteria;c__Alphaproteobacteria;o__Rhizobiales;f__Bradyrh	0	0
L6	k__Bacteria;p__Proteobacteria;c__Alphaproteobacteria;o__Rhizobiales;f__Bradyrh	0	0
L6	k__Bacteria;p__Proteobacteria;c__Alphaproteobacteria;o__Rhizobiales;f__Brucella	0	0
L6	k__Bacteria;p__Proteobacteria;c__Alphaproteobacteria;o__Rhizobiales;f__Brucella	0	0
L6	k__Bacteria;p__Proteobacteria;c__Alphaproteobacteria;o__Rhizobiales;f__Hyphon	0	0
L6	k__Bacteria;p__Proteobacteria;c__Alphaproteobacteria;o__Rhizobiales;f__Methylo	0	0
L6	k__Bacteria;p__Proteobacteria;c__Alphaproteobacteria;o__Rhizobiales;f__Methylo	0	0
L6	k__Bacteria;p__Proteobacteria;c__Alphaproteobacteria;o__Rhizobiales;f__Phyllob	0	0
L6	k__Bacteria;p__Proteobacteria;c__Alphaproteobacteria;o__Rhizobiales;f__Phyllob	0	0
L6	k__Bacteria;p__Proteobacteria;c__Alphaproteobacteria;o__Rhizobiales;f__Phyllob	0	0
L6	k__Bacteria;p__Proteobacteria;c__Alphaproteobacteria;o__Rhizobiales;f__Rhizobi	0	0
L6	k__Bacteria;p__Proteobacteria;c__Alphaproteobacteria;o__Rhizobiales;f__Rhizobi	0	0
L6	k__Bacteria;p__Proteobacteria;c__Alphaproteobacteria;o__Rhizobiales;f__Xanthol	0	0
L6	k__Bacteria;p__Proteobacteria;c__Alphaproteobacteria;o__Rhodobacterales;f__Rh	0	0
L6	k__Bacteria;p__Proteobacteria;c__Alphaproteobacteria;o__Rhodobacterales;f__Rh	0	0
L6	k__Bacteria;p__Proteobacteria;c__Alphaproteobacteria;o__Rhodobacterales;f__Rh	0	0
L6	k__Bacteria;p__Proteobacteria;c__Alphaproteobacteria;o__Rhodobacterales;f__Rh	0	0
L6	k__Bacteria;p__Proteobacteria;c__Alphaproteobacteria;o__Rhodospirillales;f__Ace	0	0
L6	k__Bacteria;p__Proteobacteria;c__Alphaproteobacteria;o__Rhodospirillales;f__Ace	0	0
L6	k__Bacteria;p__Proteobacteria;c__Alphaproteobacteria;o__Rickettsiales;f__;g__	0	0
L6	k__Bacteria;p__Proteobacteria;c__Alphaproteobacteria;o__Rickettsiales;f__Rickett	0	0
L6	k__Bacteria;p__Proteobacteria;c__Alphaproteobacteria;o__Rickettsiales;f__mitoch	0	0
L6	k__Bacteria;p__Proteobacteria;c__Alphaproteobacteria;o__Rickettsiales;f__mitoch	0	0
L6	k__Bacteria;p__Proteobacteria;c__Alphaproteobacteria;o__Rickettsiales;f__mitoch	0	0
L6	k__Bacteria;p__Proteobacteria;c__Alphaproteobacteria;o__Rickettsiales;f__mitoch	0	0
L6	k__Bacteria;p__Proteobacteria;c__Alphaproteobacteria;o__Rickettsiales;f__mitoch	0	0
L6	k__Bacteria;p__Proteobacteria;c__Alphaproteobacteria;o__Sphingomonadales;f__	0	0
L6	k__Bacteria;p__Proteobacteria;c__Alphaproteobacteria;o__Sphingomonadales;f__	0	0
L6	k__Bacteria;p__Proteobacteria;c__Alphaproteobacteria;o__Sphingomonadales;f__	0	0
L6	k__Bacteria;p__Proteobacteria;c__Alphaproteobacteria;o__Sphingomonadales;f__	0	0
L6	k__Bacteria;p__Proteobacteria;c__Alphaproteobacteria;o__Sphingomonadales;f__	0	0
L6	k__Bacteria;p__Proteobacteria;c__Betaproteobacteria;o__;f__;g__	0	0
L6	k__Bacteria;p__Proteobacteria;c__Betaproteobacteria;o__Burkholderiales;f__;g__	0	0
L6	k__Bacteria;p__Proteobacteria;c__Betaproteobacteria;o__Burkholderiales;f__Alca	0	0
L6	k__Bacteria;p__Proteobacteria;c__Betaproteobacteria;o__Burkholderiales;f__Alca	0	0
L6	k__Bacteria;p__Proteobacteria;c__Betaproteobacteria;o__Burkholderiales;f__Alca	0	0
L6	k__Bacteria;p__Proteobacteria;c__Betaproteobacteria;o__Burkholderiales;f__Alca	0	0
L6	k__Bacteria;p__Proteobacteria;c__Betaproteobacteria;o__Burkholderiales;f__Alca	0	0
L6	k__Bacteria;p__Proteobacteria;c__Betaproteobacteria;o__Burkholderiales;f__Alca	0.21	0.22
L6	k__Bacteria;p__Proteobacteria;c__Betaproteobacteria;o__Burkholderiales;f__Burk	0	0
L6	k__Bacteria;p__Proteobacteria;c__Betaproteobacteria;o__Burkholderiales;f__Burk	0	0
L6	k__Bacteria;p__Proteobacteria;c__Betaproteobacteria;o__Burkholderiales;f__Burk	0	0
L6	k__Bacteria;p__Proteobacteria;c__Betaproteobacteria;o__Burkholderiales;f__Com	0.36	0.39
L6	k__Bacteria;p__Proteobacteria;c__Betaproteobacteria;o__Burkholderiales;f__Com	0	0

L6	k__Bacteria;p__Proteobacteria;c__Betaproteobacteria;o__Burkholderiales;f__Com	0	0
L6	k__Bacteria;p__Proteobacteria;c__Betaproteobacteria;o__Burkholderiales;f__Com	0	0
L6	k__Bacteria;p__Proteobacteria;c__Betaproteobacteria;o__Burkholderiales;f__Com	0	0
L6	k__Bacteria;p__Proteobacteria;c__Betaproteobacteria;o__Burkholderiales;f__Com	0	0
L6	k__Bacteria;p__Proteobacteria;c__Betaproteobacteria;o__Burkholderiales;f__Com	0.04	0.01
L6	k__Bacteria;p__Proteobacteria;c__Betaproteobacteria;o__Burkholderiales;f__Com	0	0
L6	k__Bacteria;p__Proteobacteria;c__Betaproteobacteria;o__Burkholderiales;f__Com	0	0
L6	k__Bacteria;p__Proteobacteria;c__Betaproteobacteria;o__Burkholderiales;f__Com	0	0
L6	k__Bacteria;p__Proteobacteria;c__Betaproteobacteria;o__Burkholderiales;f__Com	0.14	0.01
L6	k__Bacteria;p__Proteobacteria;c__Betaproteobacteria;o__Burkholderiales;f__Com	0	0
L6	k__Bacteria;p__Proteobacteria;c__Betaproteobacteria;o__Burkholderiales;f__Com	0	0
L6	k__Bacteria;p__Proteobacteria;c__Betaproteobacteria;o__Burkholderiales;f__Com	0	0
L6	k__Bacteria;p__Proteobacteria;c__Betaproteobacteria;o__Burkholderiales;f__Com	0	0
L6	k__Bacteria;p__Proteobacteria;c__Betaproteobacteria;o__Burkholderiales;f__Com	0	0
L6	k__Bacteria;p__Proteobacteria;c__Betaproteobacteria;o__Burkholderiales;f__Com	0	0
L6	k__Bacteria;p__Proteobacteria;c__Betaproteobacteria;o__Burkholderiales;f__Oxal	0	0
L6	k__Bacteria;p__Proteobacteria;c__Betaproteobacteria;o__Burkholderiales;f__Oxal	0	0.01
L6	k__Bacteria;p__Proteobacteria;c__Betaproteobacteria;o__Burkholderiales;f__Oxal	0	0
L6	k__Bacteria;p__Proteobacteria;c__Betaproteobacteria;o__Burkholderiales;f__Oxal	0	0
L6	k__Bacteria;p__Proteobacteria;c__Betaproteobacteria;o__Burkholderiales;f__Oxal	0	0
L6	k__Bacteria;p__Proteobacteria;c__Betaproteobacteria;o__Burkholderiales;f__Oxal	0	0
L6	k__Bacteria;p__Proteobacteria;c__Betaproteobacteria;o__Ellin6067;f__;g__	0	0
L6	k__Bacteria;p__Proteobacteria;c__Betaproteobacteria;o__Methylophilales;f__Met	0	0
L6	k__Bacteria;p__Proteobacteria;c__Betaproteobacteria;o__Methylophilales;f__Met	0	0
L6	k__Bacteria;p__Proteobacteria;c__Betaproteobacteria;o__Neisseriales;f__Neisseri	0	0
L6	k__Bacteria;p__Proteobacteria;c__Betaproteobacteria;o__Neisseriales;f__Neisseri	0	0
L6	k__Bacteria;p__Proteobacteria;c__Betaproteobacteria;o__Neisseriales;f__Neisseri	0	0
L6	k__Bacteria;p__Proteobacteria;c__Betaproteobacteria;o__Neisseriales;f__Neisseri	0	0
L6	k__Bacteria;p__Proteobacteria;c__Betaproteobacteria;o__Rhodocyclales;f__Rhod	0	0
L6	k__Bacteria;p__Proteobacteria;c__Betaproteobacteria;o__Rhodocyclales;f__Rhod	0	0
L6	k__Bacteria;p__Proteobacteria;c__Betaproteobacteria;o__Rhodocyclales;f__Rhod	0	0
L6	k__Bacteria;p__Proteobacteria;c__Betaproteobacteria;o__Rhodocyclales;f__Rhod	0	0
L6	k__Bacteria;p__Proteobacteria;c__Betaproteobacteria;o__Rhodocyclales;f__Rhod	0	0
L6	k__Bacteria;p__Proteobacteria;c__Betaproteobacteria;o__Rhodocyclales;f__Rhod	0	0
L6	k__Bacteria;p__Proteobacteria;c__Betaproteobacteria;o__Rhodocyclales;f__Rhod	0	0
L6	k__Bacteria;p__Proteobacteria;c__Betaproteobacteria;o__SBla14;f__;g__	0	0
L6	k__Bacteria;p__Proteobacteria;c__Betaproteobacteria;o__Tremblayales;f__;g__	0	0
L6	k__Bacteria;p__Proteobacteria;c__Deltaproteobacteria;o__Desulfovibrionales;f__D	0	0.02
L6	k__Bacteria;p__Proteobacteria;c__Deltaproteobacteria;o__Desulfovibrionales;f__D	0.63	0.14
L6	k__Bacteria;p__Proteobacteria;c__Deltaproteobacteria;o__Desulfovibrionales;f__D	1.06	0.2
L6	k__Bacteria;p__Proteobacteria;c__Deltaproteobacteria;o__GMD14H09;f__;g__	0	0.02
L6	k__Bacteria;p__Proteobacteria;c__Deltaproteobacteria;o__Myxococcales;f__;g__	0	0
L6	k__Bacteria;p__Proteobacteria;c__Deltaproteobacteria;o__Myxococcales;f__0319	0	0
L6	k__Bacteria;p__Proteobacteria;c__Epsilonproteobacteria;o__Campylobacterales;f_	0	0
L6	k__Bacteria;p__Proteobacteria;c__Epsilonproteobacteria;o__Campylobacterales;f_	0.9	0.76
L6	k__Bacteria;p__Proteobacteria;c__Epsilonproteobacteria;o__Campylobacterales;f_	0	0
L6	k__Bacteria;p__Proteobacteria;c__Epsilonproteobacteria;o__Campylobacterales;f_	0	0
L6	k__Bacteria;p__Proteobacteria;c__Epsilonproteobacteria;o__Campylobacterales;f_	0	0
L6	k__Bacteria;p__Proteobacteria;c__Gammaproteobacteria;o__Aeromonadales;f__A	0.01	0
L6	k__Bacteria;p__Proteobacteria;c__Gammaproteobacteria;o__Aeromonadales;f__S	0	0

L6	k__Bacteria;p__Proteobacteria;c__Gammaproteobacteria;o__Aeromonadales;f__S	0	0
L6	k__Bacteria;p__Proteobacteria;c__Gammaproteobacteria;o__Aeromonadales;f__S	0	0
L6	k__Bacteria;p__Proteobacteria;c__Gammaproteobacteria;o__Aeromonadales;f__S	0	0
L6	k__Bacteria;p__Proteobacteria;c__Gammaproteobacteria;o__Aeromonadales;f__S	0.01	0.01
L6	k__Bacteria;p__Proteobacteria;c__Gammaproteobacteria;o__Alteromonadales;f__	0	0
L6	k__Bacteria;p__Proteobacteria;c__Gammaproteobacteria;o__Alteromonadales;f__	0	0
L6	k__Bacteria;p__Proteobacteria;c__Gammaproteobacteria;o__Alteromonadales;f__	0	0
L6	k__Bacteria;p__Proteobacteria;c__Gammaproteobacteria;o__Cardiobacteriales;f__	0	0
L6	k__Bacteria;p__Proteobacteria;c__Gammaproteobacteria;o__Cardiobacteriales;f__	0	0
L6	k__Bacteria;p__Proteobacteria;c__Gammaproteobacteria;o__Enterobacteriales;f_	2.14	0.29
L6	k__Bacteria;p__Proteobacteria;c__Gammaproteobacteria;o__Enterobacteriales;f_	0	0
L6	k__Bacteria;p__Proteobacteria;c__Gammaproteobacteria;o__Enterobacteriales;f_	0	0
L6	k__Bacteria;p__Proteobacteria;c__Gammaproteobacteria;o__Enterobacteriales;f_	0	0
L6	k__Bacteria;p__Proteobacteria;c__Gammaproteobacteria;o__Enterobacteriales;f_	0	0
L6	k__Bacteria;p__Proteobacteria;c__Gammaproteobacteria;o__Enterobacteriales;f_	0.02	0
L6	k__Bacteria;p__Proteobacteria;c__Gammaproteobacteria;o__Enterobacteriales;f_	0	0
L6	k__Bacteria;p__Proteobacteria;c__Gammaproteobacteria;o__Enterobacteriales;f_	0	0
L6	k__Bacteria;p__Proteobacteria;c__Gammaproteobacteria;o__Enterobacteriales;f_	0.01	0
L6	k__Bacteria;p__Proteobacteria;c__Gammaproteobacteria;o__Enterobacteriales;f_	0	0
L6	k__Bacteria;p__Proteobacteria;c__Gammaproteobacteria;o__Enterobacteriales;f_	0	0
L6	k__Bacteria;p__Proteobacteria;c__Gammaproteobacteria;o__Enterobacteriales;f_	0	0
L6	k__Bacteria;p__Proteobacteria;c__Gammaproteobacteria;o__Enterobacteriales;f_	0	0
L6	k__Bacteria;p__Proteobacteria;c__Gammaproteobacteria;o__Enterobacteriales;f_	0	0
L6	k__Bacteria;p__Proteobacteria;c__Gammaproteobacteria;o__Enterobacteriales;f_	0	0
L6	k__Bacteria;p__Proteobacteria;c__Gammaproteobacteria;o__Enterobacteriales;f_	0.01	0
L6	k__Bacteria;p__Proteobacteria;c__Gammaproteobacteria;o__Enterobacteriales;f_	0	0
L6	k__Bacteria;p__Proteobacteria;c__Gammaproteobacteria;o__Enterobacteriales;f_	0	0
L6	k__Bacteria;p__Proteobacteria;c__Gammaproteobacteria;o__Enterobacteriales;f_	0	0
L6	k__Bacteria;p__Proteobacteria;c__Gammaproteobacteria;o__Legionellales;f__;g__	0	0
L6	k__Bacteria;p__Proteobacteria;c__Gammaproteobacteria;o__Legionellales;f__Cox	0	0
L6	k__Bacteria;p__Proteobacteria;c__Gammaproteobacteria;o__Legionellales;f__Legi	0	0
L6	k__Bacteria;p__Proteobacteria;c__Gammaproteobacteria;o__Oceanospirillales;f__	0	0
L6	k__Bacteria;p__Proteobacteria;c__Gammaproteobacteria;o__Pasteurellales;f__Pa:	0.01	0
L6	k__Bacteria;p__Proteobacteria;c__Gammaproteobacteria;o__Pasteurellales;f__Pa:	0.6	0.02
L6	k__Bacteria;p__Proteobacteria;c__Gammaproteobacteria;o__Pasteurellales;f__Pa:	0	0
L6	k__Bacteria;p__Proteobacteria;c__Gammaproteobacteria;o__Pasteurellales;f__Pa:	0	0
L6	k__Bacteria;p__Proteobacteria;c__Gammaproteobacteria;o__Pasteurellales;f__Pa:	0	0
L6	k__Bacteria;p__Proteobacteria;c__Gammaproteobacteria;o__Pasteurellales;f__Pa:	2.54	0.12
L6	k__Bacteria;p__Proteobacteria;c__Gammaproteobacteria;o__Pasteurellales;f__Pa:	0	0
L6	k__Bacteria;p__Proteobacteria;c__Gammaproteobacteria;o__Pasteurellales;f__Pa:	0.08	0.43
L6	k__Bacteria;p__Proteobacteria;c__Gammaproteobacteria;o__Pseudomonadales;f_	0	0
L6	k__Bacteria;p__Proteobacteria;c__Gammaproteobacteria;o__Pseudomonadales;f_	0	0.01
L6	k__Bacteria;p__Proteobacteria;c__Gammaproteobacteria;o__Pseudomonadales;f_	0	0
L6	k__Bacteria;p__Proteobacteria;c__Gammaproteobacteria;o__Pseudomonadales;f_	0.06	0.07
L6	k__Bacteria;p__Proteobacteria;c__Gammaproteobacteria;o__Pseudomonadales;f_	0	0
L6	k__Bacteria;p__Proteobacteria;c__Gammaproteobacteria;o__Pseudomonadales;f_	0	0
L6	k__Bacteria;p__Proteobacteria;c__Gammaproteobacteria;o__Pseudomonadales;f_	0	0
L6	k__Bacteria;p__Proteobacteria;c__Gammaproteobacteria;o__Vibrionales;f__Pseuc	0	0
L6	k__Bacteria;p__Proteobacteria;c__Gammaproteobacteria;o__Vibrionales;f__Vibric	0	0
L6	k__Bacteria;p__Proteobacteria;c__Gammaproteobacteria;o__Xanthomonadales;f_	0	0

L6	k__Bacteria;p__Proteobacteria;c__Gammaproteobacteria;o__Xanthomonadales;f_	0	0.02
L6	k__Bacteria;p__Proteobacteria;c__Gammaproteobacteria;o__Xanthomonadales;f_	0	0
L6	k__Bacteria;p__Proteobacteria;c__Gammaproteobacteria;o__Xanthomonadales;f_	0	0
L6	k__Bacteria;p__Proteobacteria;c__Gammaproteobacteria;o__Xanthomonadales;f_	0	0
L6	k__Bacteria;p__Proteobacteria;c__Gammaproteobacteria;o__Xanthomonadales;f_	0	0
L6	k__Bacteria;p__Proteobacteria;c__Gammaproteobacteria;o__Xanthomonadales;f_	0	0
L6	k__Bacteria;p__SR1;c__;o__;f__;g__	0	0
L6	k__Bacteria;p__Spirochaetes;c__MVP-15;o__PL-11B10;f__;g__	0	0
L6	k__Bacteria;p__Spirochaetes;c__Spirochaetes;o__Sphaerochaetales;f__Sphaeroch	0.01	0
L6	k__Bacteria;p__Spirochaetes;c__Spirochaetes;o__Spirochaetales;f__Spirochaetace	0	0
L6	k__Bacteria;p__Spirochaetes;c__Spirochaetes;o__Spirochaetales;f__Spirochaetace	0.03	0.03
L6	k__Bacteria;p__Synergistetes;c__Synergistia;o__Synergistales;f__Dethiosulfovibric	0	0
L6	k__Bacteria;p__Synergistetes;c__Synergistia;o__Synergistales;f__Dethiosulfovibric	0	0
L6	k__Bacteria;p__Synergistetes;c__Synergistia;o__Synergistales;f__Synergistaceae;g	0	0.01
L6	k__Bacteria;p__Synergistetes;c__Synergistia;o__Synergistales;f__Synergistaceae;g	0	0
L6	k__Bacteria;p__Synergistetes;c__Synergistia;o__Synergistales;f__Synergistaceae;g	0	0
L6	k__Bacteria;p__TM6;c__SJA-4;o__;f__;g__	0	0
L6	k__Bacteria;p__TM7;c__TM7-3;o__;f__;g__	0	0
L6	k__Bacteria;p__TM7;c__TM7-3;o__CW040;f__;g__	0	0
L6	k__Bacteria;p__TM7;c__TM7-3;o__CW040;f__F16;g__	0	0
L6	k__Bacteria;p__TM7;c__TM7-3;o__EW055;f__;g__	0	0
L6	k__Bacteria;p__Tenericutes;c__Mollicutes;o__Acholeplasmatales;f__;g__	0	0
L6	k__Bacteria;p__Tenericutes;c__Mollicutes;o__Anaeroplasmatales;f__Anaeroplasm	0	0
L6	k__Bacteria;p__Tenericutes;c__Mollicutes;o__Anaeroplasmatales;f__Anaeroplasm	0	0
L6	k__Bacteria;p__Tenericutes;c__Mollicutes;o__RF39;f__;g__	0.02	0.08
L6	k__Bacteria;p__Tenericutes;c__RF3;o__ML615J-28;f__;g__	0	0
L6	k__Bacteria;p__Verrucomicrobia;c__Opitutae;o__Opitutales;f__Opitutaceae;g__	0	0
L6	k__Bacteria;p__Verrucomicrobia;c__Opitutae;o__[Cerasicoccales];f__[Cerasicocca	0	0
L6	k__Bacteria;p__Verrucomicrobia;c__Verruco-5;o__WCHB1-41;f__RFP12;g__	0	0
L6	k__Bacteria;p__Verrucomicrobia;c__Verruco-5;o__WCHB1-41;f__WCHB1-25;g__	0	0
L6	k__Bacteria;p__Verrucomicrobia;c__Verrucomicrobiae;o__Verrucomicrobiales;f__	0	0
L6	k__Bacteria;p__Verrucomicrobia;c__Verrucomicrobiae;o__Verrucomicrobiales;f__	0	0
L6	k__Bacteria;p__WPS-2;c__;o__;f__;g__	0	0
L6	k__Bacteria;p__[Thermi];c__Deinococci;o__Deinococcales;f__Deinococcaceae;g__	0	0

G0001	G0001	G0001	G0001	G0001	G0001	G0001	G0002	G0002	G0002	G0002	G0002	G0002	G0002	G0002
s003	s004	s005	s006	s007	s008	s009	s011	s012	s013	s014	s015	s016	s017	s018
0	0.17	0.17	0.04	0.01	0.02	0.01	0.01	0.01	0.01	0.01	0.01	0.01	0.01	0.01
0	0	0	0	0	0	0	0	0	0	0	0	0	0	0
0.88	1.51	2.03	4.88	0.19	0.46	0.58	0.53	0.19	1.1	0.26	0.54	0.51	0.42	0.65
44.1	43.1	43.1	31.3	52.6	53.3	38.5	38.7	54.3	51.6	57.1	38.7	46	43.8	49.4
0	0	0	0	0.04	0	0	0	0	0	0	0	0	0	0
0	0.01	0	0	0	0	0	0	0	0	0	0	0	0	0
0	0.02	0.01	0.37	0	0.01	0	0	0	0.01	0	0	0.01	0	0
0	0	0	0	0	0	0	0	0	0	0	0	0	0	0
0	0.03	0	0	0	0	0	0	0	0	0	0	0	0	0
0	0.02	0.01	0	0	0	0	0	0	0	0	0	0	0	0
25.6	39.3	37	31.2	33.5	26.1	35.7	26.1	22.5	38.3	23.8	27.9	15.3	16.4	33.3
18.6	0.58	2.92	13.6	7.25	16.2	21.6	15.4	16.6	2.69	14.8	20.4	28.1	29	12.7
0	0	0	0	0	0	0	0	0	0	0	0	0	0	0
0	0.22	0.15	0	0	0	0	0	0	0	0.01	0	0	0	0
0.01	0.56	0.48	0.01	0	0.01	0.01	0.01	0.01	0.01	0	0	0	0	0
10.8	13.3	13.2	18.5	6.36	3.87	3.53	19.2	6.28	6.18	3.98	12.3	10.1	10.3	3.94
0	0	0	0	0	0	0	0	0	0	0	0	0	0	0
0	0.29	0.25	0.02	0.02	0.03	0.02	0.04	0.04	0.02	0.02	0.03	0.01	0.01	0.01
0	0.13	0.14	0	0	0	0	0	0	0	0	0	0.01	0.01	0
0	0	0	0	0	0	0	0	0	0	0	0	0	0	0
0.01	0.08	0.05	0.01	0	0	0	0	0	0	0	0	0.01	0.01	0
0.01	0.61	0.41	0.08	0.04	0.03	0.03	0.04	0.03	0.09	0.03	0.05	0.01	0.02	0.01
0	0.09	0.06	0.01	0	0	0.01	0	0	0	0	0	0.01	0	0
0	0	0	0	0	0	0	0	0	0	0	0	0	0	0
0	0	0	0	0	0	0	0	0	0	0	0	0	0	0
0	0	0	0	0	0	0	0	0	0	0	0	0	0	0
0	0.11	0.14	0.04	0.01	0.01	0	0.01	0.01	0.01	0.01	0.01	0.01	0.01	0.01
0	0.01	0	0	0	0	0	0	0	0	0	0	0	0	0
0	0.04	0.03	0	0	0	0	0	0	0	0	0	0	0	0
0	0	0	0	0	0	0	0	0	0	0	0	0	0	0
0	0	0	0	0	0	0	0	0	0	0	0	0	0	0
0	0	0.02	0	0	0	0	0	0	0	0	0	0	0	0
0	0	0	0.01	0	0	0	0	0	0	0	0	0	0	0
0	0	0	0	0	0	0	0	0	0	0	0	0	0	0
0.04	0.03	0.14	0.66	0	0.03	0.01	0	0.01	0.03	0	0.01	0	0	0
0	0	0	0.01	0.01	0	0	0.01	0	0	0	0	0	0.01	0
0	0	0	0.01	0	0	0	0	0	0	0	0	0	0	0
0	0.01	0.01	0.02	0	0.01	0	0	0	0.01	0	0	0	0	0
0	0	0	0	0	0	0	0	0	0	0	0	0	0	0
0	0.02	0	0.03	0	0	0	0	0	0	0	0	0	0	0
0.09	0.39	0.15	2.29	0.06	0.05	0.03	0.02	0.02	0.03	0.01	0.06	0.07	0.05	0.04

0	0	0	0	0	0	0	0	0	0	0	0	0	0	0
0	0	0.01	0.01	0	0	0	0	0	0	0	0	0	0	0
0	0.01	0	0	0	0	0	0	0	0	0	0	0	0	0
0	0	0	0	0	0	0	0	0	0	0	0	0	0	0
0	0	0	0	0	0	0	0	0	0	0	0	0	0	0
0	0	0	0	0	0	0	0	0	0	0	0	0	0	0
0	0	0.01	0.02	0	0	0	0	0	0	0	0	0	0	0
0	0	0	0	0	0	0	0	0	0	0	0	0	0	0
0	0	0	0	0	0	0	0	0	0	0	0	0	0	0
0	0.01	0	0	0	0	0	0	0	0	0	0	0	0	0
0	0	0	0	0	0	0	0	0	0	0	0	0	0	0
0	0	0	0	0	0	0	0	0	0	0	0	0	0	0
0	0	0	0	0	0	0	0	0	0	0	0	0	0	0
0	0	0	0	0	0	0	0	0	0	0	0	0	0	0
0	0	0	0	0	0	0	0	0	0	0	0	0	0	0
0	0	0	0	0	0	0	0	0	0	0	0	0	0	0
0	0	0	0	0	0	0	0	0	0	0	0	0	0	0
0	0.08	0.03	0.02	0	0.01	0	0	0	0	0	0	0	0	0
0	0.01	0	0	0	0	0	0	0	0	0	0	0	0	0
0.09	0.03	0.02	0.61	0.05	0.11	0.02	0.18	0.04	0.55	0.05	0.25	0.06	0.05	0.04
0	0	0	0	0	0	0	0	0	0	0	0	0	0	0
0	0	0	0	0	0	0	0	0	0	0	0	0	0	0
0	0.02	0.02	0.03	0	0	0	0	0	0	0	0	0	0	0
0	0	0	0	0	0	0	0	0	0	0	0	0	0	0
0	0.01	0	0.02	0	0	0	0.01	0	0.02	0	0.01	0	0	0
0	0.04	0	0.01	0	0	0	0	0	0	0	0	0	0	0
0	0	0	0	0	0	0	0	0	0	0	0	0	0	0
0	0	0	0	0	0	0	0	0	0	0	0	0	0	0
0	0	0	0.01	0	0	0	0	0	0	0	0	0	0	0
0	0	0	0	0	0	0	0	0	0	0	0	0	0	0
0	0	0	0	0	0	0	0	0	0	0	0	0	0	0
0	0	0	0	0	0	0	0	0	0	0	0	0	0	0
0	0.05	0.02	0.01	0	0	0	0	0	0	0	0	0	0	0
0	0	0.01	0	0	0	0	0	0	0	0	0	0	0	0
0	0	0	0	0	0	0	0	0	0	0	0	0	0	0
0	0	0	0	0	0	0	0	0	0	0	0	0	0	0
0	0	0	0	0	0	0	0	0	0	0	0	0	0	0
0	0	0	0	0	0	0	0	0	0	0	0	0	0	0
0.53	0.02	0.52	0.12	0.02	0.04	0.02	0.26	0.06	0.18	0.11	0.03	0.35	0.28	0.47
0	0	0	0	0	0	0	0	0	0	0	0	0	0	0
0.02	0.11	0.1	0.15	0.02	0.02	0.44	0.02	0.02	0.08	0	0.01	0	0.01	0
0	0	0	0	0	0	0	0	0	0	0	0	0	0	0
0	0.02	0.01	0	0	0	0.02	0	0	0	0	0	0	0	0
0.05	0.36	0.88	0.68	0.02	0.11	0.01	0.01	0.02	0.19	0.04	0.03	0.01	0.01	0.07
0.05	0.02	0.03	0.1	0.01	0.05	0.01	0.01	0.01	0	0.01	0.12	0.01	0.01	0.01
0	0	0	0	0	0	0	0	0	0	0	0	0	0	0
0	0	0	0	0	0	0	0	0	0	0	0	0	0	0
0	0.26	0.04	0.06	0	0.01	0	0	0	0	0	0	0	0	0
0.04	4.83	3.71	0.32	0.09	0.1	0.16	0.22	2.27	1.91	0.17	0.26	0.41	0.34	0.11
0	0	0	0	0	0	0	0	0	0	0	0	0	0	0

0	0	0	0	0	0	0	0	0	0	0	0	0	0	0
0	0	0	0	0	0	0	0	0	0	0	0	0	0	0
0	0	0	0	0	0	0	0	0	0	0	0	0	0	0
13.2	3.09	5.17	21	39.1	38.1	15	25.8	32.3	6.92	34.4	25.7	12.2	12.4	17.8
0	0	0	0.01	0.01	0	0	0	0.01	0.01	0.01	0.01	0	0	0
0	0	0	0	0	0	0	0	0	0	0	0	0	0	0
0	0	0	0	0	0	0	0	0	0	0	0	0	0	0
1	3.46	2.84	1.03	1.26	1.03	2.82	2.04	1.52	1.63	1.83	1.33	0.6	0.6	1.43
0	0	0.01	0	0	0	0	0	0.02	0	0	0	0	0	0
0	0.01	0.01	0	0	0	0	0	0	0.01	0	0	0	0	0
25.9	3.75	9.27	7.56	1.86	1.57	17.6	2.37	2.62	11.6	10.2	7.14	30.1	27.6	23.6
0	0.02	0.04	0	0	0	0	0	0.01	0	0	0	0	0	0
0.7	1.79	1.47	0.13	1.68	0.08	1.13	0.21	0.07	0.54	0.15	0.17	0.22	0.24	0.27
0	0.01	0	0.03	0	0	0	0	0.04	0.08	0	0	0	0	0
1.25	13.3	10.3	0.6	0.33	3.49	0.3	0.41	0.64	27.5	6.03	2.66	1.17	1.22	0.26
0	0	0	0	0	0	0	0	0	0	0	0	0	0	0
1.46	0.83	1.27	0.16	1.61	8.13	1.18	6.65	13.4	0.6	3.55	0.57	1.07	1.27	5.87
0	0.02	0.01	0	0.01	0	0	0	0	0.09	0	0	0	0	0
0.02	1.75	1.21	0.01	0.09	0.03	0.01	0.01	0.03	0.02	0.01	0.07	0.01	0	0.01
0	0.09	0.06	0.03	4.31	0.03	0.03	0.14	0.18	0.5	0.02	0.02	0.02	0.01	0.01
0	0	0	0	0	0	0	0	0	0	0	0	0	0	0
0	0.09	0.08	0.04	0.01	0.01	0.02	0.01	0.03	0.01	0	0.01	0.01	0	0
0.45	0.97	0.67	0.17	2.14	0.51	0.11	0.76	1.16	0.2	0.52	0.31	0.13	0.12	0.02
0.03	9.02	6.98	0.18	0.05	0.21	0.15	0.04	0.02	0.06	0.24	0.39	0.04	0.01	0.08
0	0	0	0	0	0	0	0	0	0	0	0	0	0	0
0	0	0	0	0	0	0	0	0	0	0	0	0	0	0
0	0	0	0	0	0	0	0	0	0	0	0	0	0	0
0	0	0	0	0	0	0	0	0	0	0	0	0	0	0
0	0	0	0.01	0	0	0	0	0	0	0	0	0	0	0
0	0	0	0	0	0	0	0	0	0	0	0	0	0	0
0	0	0	0	0	0	0	0	0	0	0	0	0	0	0
0	0	0	0	0	0	0	0	0	0	0	0	0	0	0
0	0	0	0.01	0	0	0	0	0	0	0	0	0	0	0
0	0	0	0	0	0	0	0	0	0	0	0	0	0	0
0	0	0	0	0	0	0	0	0	0	0	0	0	0	0
0	0.01	0	0	0	0	0	0	0	0	0	0	0	0	0
0	0.12	0.02	0.01	0	0	0	0	0	0	0	0	0	0	0
0	0	0	0	0	0	0	0	0	0	0	0	0	0	0
0	0	0	0	0.04	0	0	0	0	0	0	0	0	0	0
0	0	0	0	0	0	0	0	0	0	0	0	0	0	0
0	0	0	0	0	0	0	0	0	0	0	0	0	0	0
0	0.01	0	0	0	0	0	0	0	0	0	0	0	0	0
0	0.02	0.01	0	0	0	0	0	0	0	0	0	0	0	0
0	0.01	0	0.37	0	0	0	0	0	0.01	0	0	0.01	0	0
0	0	0	0	0	0	0	0	0	0	0	0	0	0	0
0	0.03	0	0	0	0	0	0	0	0	0	0	0	0	0
0	0	0	0	0	0	0	0	0	0	0	0	0	0	0
0	0	0	0	0	0	0	0	0	0	0	0	0	0	0
0	0.02	0.01	0	0	0	0	0	0	0	0	0	0	0	0
0	0.05	0	0	0	0	0	0	0	0	0	0	0	0	0

0	0	0	0	0	0	0	0	0	0	0	0.02	0	0	0
0	0	0	0	0	0	0	0	0	0	0	0	0	0	0
0	0	0	0	0	0	0	0	0	0	0	0	0	0	0
0	0	0	0	0	0	0	0	0	0	0	0	0	0	0
0	0	0	0	0	0	0	0	0	0	0	0	0	0	0
0	0	0	0.01	0	0	0	0	0	0	0	0	0	0	0
0	0	0	0	0	0	0	0	0	0	0	0	0	0	0
0	0	0	0.01	0	0	0	0	0	0	0	0	0	0	0
0	0	0	0.01	0	0	0	0	0	0	0	0	0	0	0
0	0	0	0	0	0	0	0	0	0	0	0	0	0	0
0	0	0	0	0	0	0	0	0	0	0	0	0	0	0
0	0	0	0	0	0	0	0	0	0	0	0	0	0	0
0	0	0	0.02	0	0	0	0	0	0	0	0	0	0	0
0	0	0	0	0	0	0	0	0	0	0	0	0	0	0
0	0	0	0	0	0	0	0	0	0	0	0	0	0	0
0.01	0.11	0.08	1.35	0.01	0.03	0.01	0.01	0.02	0.03	0.01	0.03	0.02	0.02	0.01
0	0	0	0	0	0	0	0	0	0	0	0	0	0	0
0	0	0	0	0	0	0	0	0	0	0	0	0	0	0
0	0.03	0.01	0	0.01	0	0	0	0	0	0	0	0	0.01	0
0	0	0	0	0	0	0	0	0	0	0	0	0	0	0
0	0	0	0	0.02	0	0	0	0	0	0	0	0	0	0
0	0	0	0	0	0	0	0	0	0	0	0.01	0	0	0
0	0.01	0.01	0.06	0	0	0	0	0	0	0	0	0	0	0
0	0	0	0	0	0	0	0	0	0	0	0	0	0	0
0	0	0	0	0	0	0	0	0	0	0	0	0	0	0
0	0	0.02	0.03	0.01	0.01	0	0.01	0	0.01	0	0.01	0	0	0
0	0	0	0	0	0	0	0	0	0	0	0	0	0	0
0	0	0	0	0	0	0	0	0	0	0	0	0	0	0
0	0	0	0	0	0	0	0	0	0	0	0	0	0	0
0.01	0.08	0.05	0.01	0	0	0	0	0	0	0	0	0	0	0
0	0	0	0	0	0	0	0	0	0	0	0	0	0	0
0	0	0	0	0	0	0	0	0	0	0	0	0	0	0
0.02	0.25	0.02	0.44	0	0.02	0.01	0.01	0.02	0.13	0.01	0.02	0.04	0.03	0.01
0	0	0	0	0	0	0	0	0	0	0	0	0	0	0
0	0	0	0	0	0	0	0	0	0	0	0	0	0	0
4.05	6.5	7.01	0.94	7.41	1.63	8.48	2.51	6.1	1.97	3.94	2.49	3.14	2.73	8.26
0	0	0	0.01	0	0	0	0	0	0	0	0	0	0	0
0	0	0	0	0	0	0	0	0	0	0	0	0	0	0
0	0.01	0	0.02	0	0	0	0	0	0	0	0	0	0	0
0	0.02	0.01	0.1	0	0.01	0	0	0	0.01	0	0	0	0	0
0	0.01	0.01	0	0	0	0.01	0.01	0.01	0	0	0	0	0	0
0	0	0	0.01	0	0	0	0	0	0	0	0	0	0	0
1.66	0.42	1.2	3.55	0.48	0.83	0.22	1.71	0.55	1.25	0.46	1.46	0.98	1	0.83
0.01	0.1	0.09	0.07	0.02	0.01	0	0.05	0.04	0.02	0.01	0.03	0.01	0	0
0	0	0	0	0	0	0	0	0	0	0	0	0	0	0
3.24	5.96	5.99	2.59	6.32	5.57	4.73	6.36	2.77	14.6	7.05	5.06	2.1	2.34	5.82
0.02	0.94	0.7	0.16	0.09	0.47	0.13	0.19	0.1	0.09	0.18	0.18	0.35	0.51	0.05
0	0	0.01	0	0	0	0.01	0	0	0	0	0	0	0	0
0.24	1.91	1.54	1.33	0.47	0.52	0.23	0.48	0.48	0.59	0.18	0.52	0.19	0.23	0.23
0	0.01	0	0	0	0	0	0	0	0	0	0	0	0	0

0	0	0	0	0	0	0	0	0	0	0	0	0	0	0
1.71	0.35	0.98	5.33	9.67	2.44	1.63	2.04	2.83	2.44	1.43	2.28	1.5	2.07	6.86
0	0	0	0	0	0	0	0	0	0	0	0	0	0	0
0.04	0.89	0.57	0.23	0.04	0.05	0.12	0.16	0.23	0.05	0.04	0.09	0.08	0.08	0.03
0	0.01	0	0	0	0	0	0	0	0	0	0	0	0	0
0	0	0	0	0	0	0	0	0	0	0	0	0	0	0
0	0.03	0.01	0	0	0	0	0	0	0	0	0	0	0	0
0	0	0	0	0	0	0	0	0	0	0.01	0	0	0	0
0	0	0	0	0	0	0	0	0	0	0	0	0	0	0
0	0	0	0.03	0	0	0.02	0	0	0.01	0	0	0	0	0
1.83	1.08	1.14	0.94	0.99	3.22	0.52	0.88	0.49	1.77	1.21	1.5	1.99	2.05	1.51
0	0	0	0	0	0	0	0	0	0	0	0	0	0	0
0.03	0.24	0.23	0.11	0.02	0.06	0.05	0.03	0.05	0.08	0.04	0.05	0.03	0.04	0.03
0	0	0	0	0	0	0	0	0	0	0	0	0	0	0
0	0	0	0	0	0	0	0	0	0	0	0	0	0	0
0.03	0.16	0.08	0.1	0.16	0.04	0.01	0.06	0.13	0.13	0.04	0.04	0.09	0.1	0.01
0.37	0.08	0.16	0.82	0.12	0.8	0.04	0.4	0.12	0.29	0.58	0.73	0.16	0.16	0.15
0	0	0	0.05	0.01	0	0	0	0.01	0	0.01	0	0	0	0
0	0	0	0	0	0	0	0	0	0	0	0	0	0	0
0	0	0	0	0	0	0	0.01	0	0	0	0	0	0	0
0	0	0	0	0	0	0	0	0	0	0	0	0	0	0
0	0.06	0.03	0	0.01	0.05	0.02	0.04	0.08	0.01	0.04	0.02	0	0.01	0
0	0	0	0.01	0.01	0.01	0.01	0.03	0.01	0.01	0.01	0.02	0	0.01	0
1.29	0.09	0.6	2.19	0.23	1.59	0.49	0.56	0.73	0.31	1.53	5.65	0.36	0.3	0.17
0	0	0	0	0	0	0	0	0	0	0	0	0	0	0
0	0	0	0	0	0	0	0	0	0	0	0	0	0	0
0	0.23	0.22	0.12	0	0.01	0	0.01	0.03	0.09	0.01	0.01	0	0.05	0
0	0	0.01	0.01	0	0.01	0	0	0	0	0	0	0	0	0
0	0.02	0.01	0.01	0.02	0.01	0.01	0.02	0.01	0	0.01	0.02	0.01	0.01	0.01
0	0	0	0	0	0	0	0	0	0	0	0	0	0	0
0.01	0	0.02	0.14	0.02	0.03	0.01	0.01	0.03	0.03	0.01	0.01	0.01	0.01	0
0.09	0.01	0.03	0.08	0.24	0.86	0.03	0.8	0.23	0.11	0.5	0.75	0.09	0.07	0.33
1.11	12	9.09	1.93	2.42	2.43	5.62	2.2	1.36	9.08	1.56	3.35	1	0.95	0.59
0	0	0	0	0	0	0	0	0	0	0	0	0	0	0
0	0.03	0.02	0.06	0.02	0	0.18	0.01	0	0	0.01	0.02	0	0	0
0.73	0.02	0.15	0.22	0.11	0.56	0.19	0.47	0.28	0.08	0.47	0.25	0.23	0.25	0.48
0.01	0	0.01	0.01	0	0.01	0.01	0.02	0.01	0	0.01	0.01	0.01	0.01	0
0	0.24	0.22	0.17	0.01	0.03	0.01	0.04	0.03	1.88	0.01	0.08	0.01	0.01	0.01
1.06	2.17	1.97	2.72	1.16	2.15	1.39	3	2.2	0.92	3.27	1.35	0.87	0.9	0.66
0.14	1.07	0.85	0.48	0.12	0.75	0.49	0.84	0.23	0.54	0.51	0.37	0.24	0.35	0.07
0	0.07	0.04	0.03	0.02	0.04	0.03	0.04	0.03	0.01	0.01	0.02	0.01	0.02	0.01
0	0.03	0.04	0.01	0.01	0.01	3.89	0.02	0.02	0	0.01	0.02	0	0.02	0
0	0.01	0.01	0	0.02	0	0	0.01	0.02	0.01	0	0	0	0	0
0.03	0	0.03	0.01	0.01	0.02	0.1	0.01	0.01	0	0	0.01	0.06	0.09	0.13
4.37	0.27	0.18	0.09	0.13	0.17	4.55	0.07	0.33	0.12	0.07	0.09	0.08	0.09	5.59
0.01	0.01	0.01	0.05	0.8	0.03	0.48	0.02	0.05	0.02	0.02	0.02	0.01	0.02	0.28
0.55	1.54	1.26	0.17	0.22	1.02	0.08	2.53	0.67	0.1	0.24	0.14	0.05	0.09	0.06
0	0	0	0	0	0	0	0	0	0	0	0	0	0	0
0	0	0	0	0	0	0	0	0	0	0	0	0	0	0
0	0.01	0	0	0	0	0	0	0	0	0	0	0	0	0

0	0	0	0	0	0	0	0	0	0	0	0	0	0	0
2.29	0.15	0.42	0.08	1.83	0.07	0.29	0.09	1.38	0.04	0.04	0.2	1.38	1.57	0.69
0	0	0	0	0	0	0	0	0	0	0	0	0	0	0
0.03	0.23	0.3	0.06	0.01	0.1	0.09	0.02	0.01	0.09	0.04	0.01	0.02	0.02	0.01
0	0	0	0	0	0	0	0	0	0	0	0	0	0	0
0.01	0	0.01	0.02	0	0.01	0.25	0.01	0	0	0	0	0.03	0.03	0
0	0	0	0	0	0	0	0	0	0	0	0	0	0	0
0	0	0	0	0	0	0	0	0	0	0	0	0	0	0
0.02	0.06	0.03	1.36	0.01	0.02	0.01	0.01	0.01	0.04	0	0.32	0.01	0.01	0.01
0	0.01	0.01	1.67	0.01	0.02	0.01	0.01	0.01	0.01	0.01	0.08	0.01	0.01	0
0	0	0	0	0	0	0	0	0	0	0	0	0	0	0
0	0	0	0.01	0	0	0	0.03	0	0	0	0.02	0	0	0
0	0	0	0.02	0	0	0	0	0	0	0	0	0	0	0
0	0	0	0	0	0	0	0	0	0	0	0	0	0	0
0	0.01	0	0.11	0	0.01	0.03	0	0	0.03	0	0.02	0	0	0
0	0	0	0	0	0	0	0	0	0	0	0	0	0	0
0	0	0	0	0	0	0	0	0	0	0	0	0	0	0
0	0	0	0	0	0	0	0	0	0	0	0	0	0	0
0	0	0	0	0	0	0	0	0	0	0	0	0	0	0
0.06	0.13	0.15	0.09	0.02	0.01	0.03	0.02	0.34	0.14	0.01	0.02	0.01	0.01	0.01
0.14	0.35	0.26	0.02	0.01	0.01	0.42	0.02	0.09	0.11	0.01	0.09	0.01	0.01	0.02
0	0	0	0	0	0	0	0	0	0	0	0	0	0	0
0	0	0	0	0	0	0	0	0	0	0	0	0	0	0
0	0	0	0	0	0	0	0	0	0	0	0	0	0	0
0.1	0	0	0	0.08	0	0	0.01	0	0	0	0	0.01	0	0.01
0	0.04	0.02	0.01	0	0	0	0	0	0.39	0.01	0	0	0	0.01
0	0	0	0	0	0	0	0	0	0	0	0	0	0	0
0	0.03	0.03	0	0	0	0	0	0	0	0	0	0	0	0
0	0.13	0.07	0.01	0	0.03	0.07	0.01	0.02	0	0	0.01	0	0	0
0.29	0.61	0.67	0.65	0.04	0.16	0.53	0.11	0.19	0.44	0.08	0.4	0.06	0.07	0.29
0.01	0.33	0.26	0.15	0.03	0.16	0.12	0.02	0.09	0.15	0.01	0.03	0.01	0.01	0.01
0.11	0	0.03	0.43	0.09	0.49	0.02	0.15	0.06	0.03	0.33	0.25	0.06	0.08	0.02
0	0	0	0	0	0	0	0	0	0	0	0	0	0	0
18.5	0.57	2.89	13.2	7.16	15.7	21.5	15.3	16.5	2.66	14.4	20.1	28	28.9	12.6
0	0.01	0	0	0	0	0	0	0	0	0	0	0	0	0
0	0	0	0	0	0	0	0	0	0	0	0	0	0	0
0	0.06	0.02	0	0	0	0	0	0	0	0.01	0	0	0	0
0	0	0	0	0	0	0	0	0	0	0	0	0	0	0
0	0.16	0.13	0	0	0	0	0	0	0	0	0	0	0	0
0	0	0	0	0	0	0	0	0	0	0	0	0	0	0
0.01	0.56	0.48	0	0	0.01	0.01	0.01	0.01	0.01	0	0	0	0	0
0	0	0	0	0	0	0	0	0	0	0	0	0	0	0
0	0	0	0	0	0	0	0	0	0	0	0	0	0	0
0	0.01	0.01	0	0	0	0	0	0	0	0	0	0	0	0
0	0	0	0	0	0	0	0	0	0	0	0	0	0	0
0	0.03	0	0	0	0	0	0	0	0	0	0	0	0	0
0	0	0.01	0.01	0	0	0	0	0	0	0	0	0	0	0
0	0	0	0	0	0	0	0	0	0	0	0	0	0	0
0	0	0	0	0	0	0	0	0	0	0	0	0	0	0
0	0.02	0	0	0	0	0	0	0	0	0	0	0	0	0

0	0.01	0	0	0	0	0	0	0	0	0	0	0	0	0
0	0	0	0	0	0	0	0	0	0	0	0	0	0	0
0	0	0	0	0	0	0	0	0	0	0	0	0	0	0
0	0	0	0	0	0	0	0	0	0	0	0	0	0	0
0	0.29	0.03	0.02	0	0	0	0	0	0	0	0	0	0	0
0	0	0	0	0	0	0	0	0	0	0	0	0	0	0
0	0.03	0	0	0	0	0	0	0	0	0	0	0	0	0
0	0.01	0	0	0	0	0	0	0	0	0	0	0	0	0
0	0	0	0	0	0	0	0	0	0	0	0	0	0	0
0	0	0	0	0	0	0	0	0	0	0	0	0	0	0
0	0	0	0	0	0	0	0	0	0	0	0	0	0	0
0	0	0	0	0	0	0	0	0	0	0	0	0	0	0
0	0	0	0	0	0	0	0	0	0	0	0	0	0	0
0	0	0	0	0	0	0	0	0	0	0	0	0	0	0
0	0	0	0	0	0	0	0	0	0	0	0	0	0	0
0	0.01	0	0	0	0	0	0	0	0	0	0	0	0	0
0	0.01	0	0	0	0	0	0	0	0	0	0	0	0	0
0	0	0	0	0	0	0	0	0	0	0	0	0	0	0
0	0.03	0.01	0.03	0	0	0	0	0	0	0	0	0	0	0
0	0	0	0	0	0	0	0	0	0	0	0	0	0	0
0	0	0	0	0	0	0	0	0	0	0	0	0	0	0
0	0.01	0	0	0	0	0	0	0	0	0	0	0	0	0
0	0	0	0	0	0	0	0	0	0	0	0	0	0	0
0	0	0	0	0	0	0	0	0	0	0	0	0	0	0
0	0	0	0	0	0	0	0	0	0	0	0	0	0	0
0	0.05	0	0	0	0	0	0	0	0	0	0	0	0	0
0	0	0	0	0	0	0	0	0	0	0	0	0	0	0
0	0	0	0	0	0	0	0	0	0	0	0	0	0	0
0	0	0	0	0	0	0	0	0	0	0	0	0	0	0
0	0	0	0	0	0	0	0	0	0	0	0	0	0	0
0	0	0	0	0	0	0	0	0	0	0	0	0	0	0
0	0	0.01	0.04	0	0	0	0	0	0	0	0	0	0	0
0	0	0	0	0	0	0	0	0	0	0	0	0	0	0
0	0	0	0	0	0	0	0	0	0	0	0	0	0	0
0	0	0	0	0	0	0	0	0	0	0	0	0	0	0
0	0	0.01	0	0	0	0	0	0	0	0	0	0	0	0
0	0.02	0.01	0	0	0	0	0	0	0	0	0	0	0	0
0	0	0	0	0	0	0	0	0	0	0	0	0.01	0.01	0
0.01	0.13	0.09	0.08	0	0.01	0	0	0	0	0	0	0.01	0.01	0
0	0	0.01	0	0	0	0	0	0	0	0	0	0	0	0
0	0	0.01	0	0	0	0	0	0	0	0	0	0	0	0
0	0	0	0	0	0	0	0	0	0	0	0	0	0	0
0	0	0	0	0	0	0	0	0	0	0	0	0	0	0
0	0	0	0	0	0	0	0	0	0	0	0	0	0	0
1.28	0.27	1.01	0.05	0.1	0.2	0.43	0.61	1.19	0.1	0.08	0.09	0.34	0.33	1.14
0	0	0	0	0	0	0	0	0	0	0	0	0	0	0
0	0	0	0	0	0	0	0	0	0	0	0	0	0	0
0	0	0	0	0	0	0	0	0	0	0	0	0	0	0
2.64	7.5	5.93	4.2	0.07	0.29	0.27	0.07	0.08	0.03	0.05	0.14	3.64	3.56	0.33
0	0	0	0	0	0	0	0	0	0	0	0	0.01	0	0

0	0	0	0	0	0	0	0	0	0	0	0	0	0	0
0	0	0	0	0	0	0	0	0	0	0	0	0	0	0
0.03	0	0	0	0	0	0	0	0	0	0	0	0.02	0.02	0
0.01	0	0	0	0	0	0	0	0	0	0	0	0.01	0.02	0
0.19	0.01	0.02	0	0	0.01	0.01	0	0.01	0	0	0	0.31	0.3	0.02
0	0	0	0	0	0	0	0	0	0	0	0	0	0	0
0.01	0	0	0	0	0	0	0	0	0	0	0	0.01	0	0
0.01	0	0	0	0	0	0	0	0	0	0	0	0.02	0.01	0
0.81	0.04	0.09	0.01	0.01	0.01	0.02	0.01	0.01	0.01	0.01	0.02	1.32	1.61	0.1
0	0	0	0	0	0	0	0	0	0	0	0	0	0	0
0.01	0	0	0	0	0	0	0	0	0	0	0	0.01	0.01	0
0	0	0	0	0	0	0	0	0	0	0	0	0	0	0
0	0	0	0	0	0	0	0	0	0	0	0	0	0	0
0	0	0	0	0	0	0	0	0	0	0	0	0	0	0
0	0	0	0	0	0	0	0	0	0	0	0	0	0	0
0	0.03	0.02	0.01	0	0	0	0	0	0	0	0	0	0	0
0	0.14	0.11	0.09	0	0	0.01	0	0	0	0	0	0	0	0
0	0	0	0	0	0	0	0	0	0	0	0	0	0	0
0	0	0	0	0	0	0	0	0	0	0	0	0	0	0
0	0	0	0	0	0	0	0	0	0	0	0	0	0	0
0	0	0	0	0	0	0	0	0	0	0	0	0	0	0
0	0	0	0	0	0	0	0	0	0	0	0	0	0	0
0	0	0	0	0	0	0	0	0	0	0	0	0	0	0
0	0	0	0	0	0	0	0	0	0	0	0	0	0	0
0	0.03	0	0.01	0	0	0	0	0	0	0	0	0	0	0
0	0	0	0	0	0	0	0	0	0	0	0	0	0	0
0	0	0	0	0	0	0	0	0	0	0	0	0	0	0
0	0	0	0	0	0	0	0	0	0	0	0	0	0	0
0.01	0	0	0	0	0	0	0	0	0	0	0	0	0.01	0
0	0	0	0	0	0	0	0	0	0	0	0	0	0	0
0	0	0	0	0	0	0	0	0	0	0	0	0	0	0
0	0	0	0	0	0	0	0	0	0	0	0	0	0	0
0	0	0	0	0	0	0	0	0	0	0	0	0	0	0
0	0	0	0	0	0	0	0	0	0	0	0	0	0	0
0	0	0	0	0	0	0	0	0	0	0	0	0	0	0
0	0	0	0	0	0	0	0	0	0	0	0	0	0	0
0	0.01	0.01	0	0.05	0	0	0	0	0	0	0	0	0	0
0.02	0.29	0.2	0.05	0	0	0.03	0.01	0.01	0.06	0.02	0.05	0.01	0	0.03
0.64	0.06	0.13	0.06	0.8	0.55	0.3	0.04	0.07	0.01	0.03	0.02	0.2	0.18	0.14
2.34	1.57	1.55	0.62	1.04	0.54	1.14	0.19	0.68	2.06	1.29	0.15	1.34	1.18	1.11
0	0.31	0.19	0.3	0	0	0	0.02	0	0.04	0	0.01	0	0	0
0	0.01	0	0	0	0	0	0	0	0	0	0	0	0	0
0	0	0	0	0	0	0	0	0	0	0	0	0	0	0
0	0	0	0	0	0	0	0	0	0	0	0	0	0	0
0.03	0.27	2.43	1.84	0.63	0.94	0.1	16.1	0.31	0.81	0.15	10.9	0.08	0.1	0.3
0	0	0	0	0	0	0	0	0	0	0	0	0	0	0
0	0	0	0	0	0	0	0	0	0	0	0	0	0	0
0	0.05	0.02	0	0	0	0	0	0	0	0	0	0	0	0
0	0	0	0	0	0	0	0	0	0	0	0	0	0	0
0	0	0	0	0	0	0	0	0.01	0	0	0	0	0	0

0	0	0	0	0.01	0	0	0	0	0	0	0	0	0	0
0	0	0	0	0	0	0	0	0	0	0	0	0	0	0
0	0	0	0	0	0	0	0	0	0	0	0	0	0	0
0.01	0	0	0.02	0.01	0.01	0.01	0.02	0.01	0.01	0.02	0.02	0.01	0.01	0.01
0	0	0	0	0	0	0	0	0	0	0	0	0	0	0
0	0	0	0	0	0	0	0	0	0	0	0	0	0	0
0	0	0	0	0	0	0	0	0	0	0	0	0	0	0
0	0	0	0	0	0	0	0	0	0	0	0	0	0	0
0	0	0	0	0	0	0	0	0	0	0	0	0	0	0
1.6	0.9	0.55	8.94	0.23	0.61	0.98	0.35	2.24	2.78	0.32	0.36	2.19	2.36	0.18
0	0	0	0	0	0	0	0	0	0	0	0	0	0	0
0	0	0	0	0	0	0	0	0	0	0	0	0	0	0
0	0	0	0	0	0	0	0	0	0	0	0	0	0	0
0	0	0	0.01	0	0	0	0	0	0	0	0	0	0	0
0	0	0.02	0.02	0	0	0	0	0.04	0	0	0	0	0	0
0	0	0	0	0	0	0	0	0	0	0	0	0	0	0
0	0	0	0	0	0	0	0	0	0	0	0	0	0	0
0	0	0	0	0	0	0	0	0	0	0	0	0	0	0
0	0	0	0.01	0	0	0	0	0	0	0	0	0	0	0
0	0	0	0	0	0	0	0	0	0	0	0	0	0	0
0	0	0	0.45	0	0.01	0	0.01	0	0	0	0	0	0	0
0	0	0	0	0	0	0	0	0	0	0	0	0	0	0
0	0	0	0	0	0	0	0	0	0	0	0	0	0	0
0	0	0	0	0	0	0	0	0	0	0	0	0	0	0
0.01	0	0	0.03	0	0	0	0	0.01	0.01	0	0	0.01	0	0
0	0	0	0	0	0	0	0	0	0	0	0	0	0	0
0	0	0	0	0	0	0	0	0	0	0	0	0	0	0
0	0	0	0	0	0	0	0	0	0	0	0	0	0	0
0	0	0	0	0	0	0	0	0	0	0	0	0	0	0
0	0	0	0	0	0	0	0	0	0	0	0	0	0	0
0	0	0	0	0	0	0	0	0	0	0	0	0	0	0
0	0	0	0	0	0	0	0	0	0	0	0	0	0	0
0.02	0.01	0	0.01	0.06	0.01	0	0.03	0.06	0	0.02	0.01	0.01	0.01	0.01
0.04	0.01	0.03	0.05	0.27	0.03	0.02	0.25	0.18	0.02	0.81	0.05	0.06	0.07	0.04
0	0	0	0	0.01	0	0	0	0	0	0	0	0	0	0
0	0	0	0	0	0	0	0	0	0	0	0	0	0	0
0	0	0	0	0	0	0	0	0	0	0	0	0	0	0
0.57	0.24	0.14	0.71	2.83	0.4	0.11	1.03	1.16	0.09	0.92	0.25	0.21	0.19	0.4
0	0	0	0	0	0	0	0	0	0	0	0	0	0	0
0.43	0.04	0.06	0.11	0.14	0.13	0.04	0.3	0.17	0.09	0.18	0.21	0.2	0.23	0.08
0	0	0	0	0	0	0	0	0	0	0	0	0	0	0
0	0.14	0.1	0.24	0	0.01	0	0	0	0.02	0	0.01	0	0	0.01
0	0	0	0	0	0	0	0	0	0	0	0	0	0	0
0.03	0	0.03	0.13	0.06	0.05	0.01	0.09	0.02	0.01	0.07	0.08	0.02	0.03	0.02
0	0	0	0	0	0	0	0	0	0	0	0	0	0	0
0	0.02	0	0	0	0	0	0	0	0	0	0	0	0	0
0	0.01	0.01	0.02	0	0	0	0	0	0	0	0	0	0	0
0	0	0	0	0	0	0	0	0	0	0	0	0	0	0
0	0	0	0	0	0	0	0	0	0	0	0	0	0	0
0	0.06	0.02	0	0	0	0	0	0	0	0	0	0	0	0

0	0.48	0.31	0.28	0	0.03	0.02	0	0	0	0	0.01	0	0	0
0	0	0	0	0	0	0	0	0	0	0	0	0	0	0
0	0	0	0	0	0	0	0	0	0	0	0	0	0	0
0	0	0	0	0	0	0	0	0	0	0	0	0	0	0
0	0.06	0.01	0.01	0	0	0	0	0	0	0	0	0	0	0
0.02	0	0	0	0	0	0	0	0	0	0	0	0	0	0
0	0	0	0	0	0	0	0	0	0	0	0	0	0	0
0	0	0	0	0	0	0	0	0	0	0	0	0	0	0
0	0.04	0.04	0	0.01	0.01	0	0.01	0	0	0	0.01	0	0	0
0	0	0	0	0	0	0	0	0	0	0	0	0	0	0
0	0.25	0.2	0.02	0.01	0.02	0.02	0.03	0.04	0.01	0.02	0.03	0.01	0.01	0.01
0	0.02	0.01	0	0	0	0	0	0	0	0	0	0	0.01	0
0	0.06	0.07	0	0	0	0	0	0	0	0	0	0	0	0
0	0.03	0	0	0	0	0	0	0	0	0	0	0	0	0
0	0	0	0	0	0	0	0	0	0	0	0	0	0	0
0	0.02	0.06	0	0	0	0	0	0	0	0	0	0.01	0	0
0	0	0	0	0	0	0	0	0	0	0	0	0	0	0
0	0	0	0	0	0	0	0	0	0	0	0	0	0	0
0.01	0	0	0	0	0	0	0	0	0	0	0	0.01	0.01	0
0	0.07	0.05	0	0	0	0	0	0	0	0	0	0	0	0
0	0	0	0	0	0	0	0	0	0	0	0	0	0	0
0	0	0	0	0	0	0	0	0	0	0	0	0	0	0
0	0	0	0	0	0	0	0	0	0	0	0	0	0	0
0	0	0	0	0	0	0	0	0	0	0	0	0	0	0
0.01	0.61	0.41	0.08	0.04	0.03	0.03	0.04	0.03	0.09	0.03	0.04	0.01	0.02	0.01
0	0	0	0	0	0	0	0	0	0	0	0	0	0	0
0	0	0	0	0	0	0	0	0	0	0	0	0	0	0
0	0	0	0	0	0	0	0	0	0	0	0	0	0	0
0	0.09	0.06	0.01	0	0	0	0	0	0	0	0	0.01	0	0
0	0	0	0	0	0	0	0	0	0	0	0	0	0	0
0	0	0	0	0	0	0	0	0	0	0	0	0	0	0
0	0	0	0	0	0	0	0	0	0	0	0	0	0	0
0	0	0	0	0	0	0	0	0	0	0	0	0	0	0
0	0	0	0	0	0	0	0	0	0	0	0	0	0	0

G0002	G0002	G0003	G0003	G0003	G0003	G0003	G0003	G0003	G0003	G0003_T2
s019	s020	s021	s022	s023	s024	s025	s026	s027	s028	s029
0	2.5	0.01	0.01	0.01	0.04	0.01	0.01	0.02	0.01	0.01
0	0	0	0	0	0	0	0	0	0	0
0.1	0.57	0.27	0.12	0.23	0.19	0.5	1.01	0.51	0.34	0.53
45	21.4	32	47.2	41.6	49.6	43	50.2	59.4	29.8	64.3
0	0	0	0	0	0	0	0	0	0	0
0	0	0	0	0	0.01	0	0	0	0	0
0	0.07	0	0	0	0.03	0.01	0	0	0	0
0	0	0	0	0	0	0	0	0	0	0
0	0.01	0	0	0	0	0.03	0	0.02	0.01	0
0	0.01	0	0	0.01	0.12	0	0	0	0	0
15.7	62.6	15.8	16.7	15.7	20.9	19.5	28.5	21.9	23.8	24.9
25.6	0.84	41.1	22.4	35.2	23.9	21.8	16.5	11.1	41.7	7.67
0	0	0	0	0	0	0	0	0	0	0
0	0.04	0	0	0	0.02	0	0.02	0	0.02	0
0.01	0.07	0	0	0	0	0	0	0.01	0	0
13.5	5.91	10.8	13.6	6.96	4.94	15	3.55	6.92	4.28	2.48
0	0	0	0	0	0.03	0	0	0	0	0
0.02	3.32	0.03	0.01	0.15	0.09	0.02	0.02	0.03	0.01	0.02
0	0.03	0	0	0	0	0	0.22	0	0	0
0	0	0	0	0	0	0	0	0	0	0
0	0.02	0.02	0	0	0.01	0	0	0	0	0
0.02	2.36	0.04	0.07	0.08	0.06	0.07	0.01	0.02	0.04	0.03
0.02	0.23	0	0	0.02	0.03	0.01	0	0	0	0
0	0	0	0	0	0.01	0	0	0	0	0
0	0	0	0	0	0	0	0	0	0	0
0	0	0	0	0	0	0	0	0	0	0
0	2.46	0	0	0.01	0.02	0.01	0.01	0.02	0.01	0.01
0	0	0	0	0	0	0	0	0	0	0
0	0.04	0	0	0	0.02	0	0	0	0	0
0	0	0	0	0	0	0	0	0	0	0
0	0	0	0	0	0	0	0	0	0	0
0	0	0	0	0	0	0	0	0	0	0
0	0	0	0	0	0	0	0	0	0	0
0	0	0	0	0	0	0	0	0	0	0
0	0.01	0	0	0	0	0.01	0	0	0	0
0	0	0	0	0	0.01	0	0	0	0	0
0	0	0	0	0	0	0	0	0	0	0
0	0	0	0	0	0	0	0	0	0	0
0	0	0	0	0	0	0	0	0	0	0
0	0	0	0	0	0	0	0	0	0	0
0.02	0.01	0.06	0.05	0.15	0.01	0.26	0.03	0.08	0.02	0.02

0	0	0	0	0	0	0	0	0	0	0
0	0	0	0	0	0	0	0	0	0	0
0	0	0	0	0	0	0	0	0	0	0
0	0	0	0	0	0	0	0	0	0	0
0	0	0	0	0	0	0	0	0	0	0
0	0	0	0	0	0	0	0	0	0	0
0	0	0	0	0	0	0	0	0	0	0
0	0	0	0	0	0	0	0	0	0	0
0	0	0	0	0	0	0	0	0	0	0
0	0	0	0	0	0	0	0	0	0	0
0	0	0	0	0	0	0	0	0	0	0
0	0	0	0	0	0	0	0	0	0	0
0	0	0	0	0	0	0	0	0	0	0
0	0	0	0	0	0	0	0	0	0	0
0	0	0	0	0	0	0	0	0	0	0
0	0	0	0	0	0	0	0	0	0	0
0	0	0	0	0	0	0	0	0	0	0
0	0	0	0	0	0	0	0	0	0	0
0	0	0	0	0	0	0	0	0	0	0
0.03	0.01	0.06	0.02	0.03	0.04	0.1	0.03	0.24	0.03	0.09
0	0	0	0	0	0	0	0	0	0	0
0	0	0	0	0	0	0	0	0	0	0
0	0	0	0	0	0	0	0	0	0	0
0	0	0	0	0	0	0	0	0	0	0
0	0	0	0	0	0	0	0	0.01	0	0
0	0	0	0	0	0	0	0	0	0	0
0	0	0	0	0	0	0	0	0	0	0
0	0	0	0	0	0	0	0	0	0	0
0	0	0	0	0	0	0	0	0	0	0
0	0	0	0	0	0	0	0	0	0	0
0	0	0	0	0	0	0	0	0	0	0
0	0	0	0	0	0	0	0	0	0	0
0	0	0	0	0	0	0	0	0	0	0
0	0	0	0	0	0	0	0	0	0	0
0	0	0	0	0	0	0	0	0	0	0
0	0	0	0	0	0	0	0	0	0	0
0	0	0	0	0	0	0	0	0	0	0
0	0	0	0	0	0	0	0	0	0	0
0.02	0.06	0.08	0.01	0.02	0.06	0.01	0.11	0.03	0.21	0.31
0	0	0	0	0	0	0	0	0	0	0
0	0.42	0.01	0.01	0.02	0.01	0.04	0.8	0.13	0.01	0.01
0	0	0	0	0	0	0	0	0	0	0
0	0	0	0	0	0	0	0	0	0	0
0.01	0.05	0.05	0.02	0	0.01	0.07	0.04	0	0.05	0.01
0.01	0	0	0	0	0.02	0	0	0	0.01	0.08
0	0	0	0	0	0	0	0	0	0	0
0	0	0	0	0	0	0	0	0	0	0
0	0	0	0	0	0	0	0	0	0	0
0.2	1.59	0.15	0.35	0.31	0.42	0.25	0.28	0.75	0.39	0.41
0	0	0	0	0	0	0	0	0	0	0

0	0	0	0	0	0	0	0	0	0	0
0	0	0	0	0	0	0	0	0	0	0
0	0	0	0	0	0.09	0	0	0	0	0
36.3	8.94	12.9	32.2	33.4	44.6	39.1	20.6	40	18.9	19.5
0	0	0	0.03	0	0	0	0	2.81	0.01	0
0	0	0	0.02	0	0	0	0	0.08	0	0
0	0	0	0	0	0	0	0	0	0	0
3.47	0.71	0.7	0.37	0.95	0.5	0.33	1.75	0.92	2.75	1.06
0	0.2	0	0	0.01	0	0	0	0.02	0	0
0	0	0	0	0	0	0	0	0	0	0
1.28	3.99	17	7.26	0.75	1.98	1.71	26.8	1.71	5.28	35.8
0	0.05	0	0	0	0.02	0	0	0.01	0	0
0.31	0.05	0.15	0.02	0.11	0.04	0.02	0.02	0.31	0.06	0.08
0	0	0	0	0	0	0	0	0	0	0.02
1.03	3.71	0.28	0.34	0.19	0.41	0.31	0.25	1.36	0.64	4.55
0	0	0	0	0	0	0	0	0	0	0
2.01	0.17	0.52	6.35	1.94	0.88	0.83	0.24	10.6	1.64	1.85
0	0	0	0.01	0	0	0	0	0.01	0	0
0.01	0.16	0.02	0.02	0.03	0.02	0.03	0.02	0.02	0.04	0
0.04	0.08	0.03	0.01	0.09	0.15	0.26	0.01	0.11	0.03	0.03
0	0	0	0	0	0	0	0	0	0	0
0.01	0.81	0	0	0.12	0.03	0.02	0.01	0	0	0.01
0.12	0.39	0.07	0.14	3.67	0.39	0.1	0.13	0.46	0.05	0.93
0.19	0.57	0.17	0.1	0.03	0.06	0.02	0.06	0.27	0.01	0.05
0	0	0	0	0	0	0	0	0	0	0
0	0	0	0	0	0	0	0	0	0	0
0	0	0	0	0	0	0	0	0	0	0
0	0	0	0	0	0	0	0	0	0	0
0	0	0	0	0	0	0	0	0	0	0
0	0	0	0	0	0	0	0	0	0	0
0	0	0	0	0	0	0	0	0	0	0
0	0	0	0	0	0	0	0	0	0	0
0	0	0	0	0	0	0	0	0	0	0
0	0	0	0	0	0	0	0	0	0	0
0	0	0	0	0	0	0	0	0	0	0
0	0	0	0	0	0	0	0	0	0	0
0	0	0	0	0	0	0	0	0	0	0
0	0	0	0	0	0	0	0	0	0	0
0	0	0	0	0	0	0	0	0	0	0
0	0	0	0	0	0.01	0	0	0	0	0
0	0	0	0	0	0	0	0	0	0	0
0	0	0	0	0	0	0	0	0	0	0
0	0.06	0	0	0	0.03	0.01	0	0	0	0
0	0	0	0	0	0	0	0	0	0	0
0	0	0	0	0	0	0	0	0	0	0
0	0.01	0	0	0	0	0.03	0	0.02	0.01	0
0	0	0	0	0	0	0	0	0	0	0
0	0	0	0	0	0	0	0	0	0	0
0	0.01	0	0	0.01	0.12	0	0	0	0	0
0	0	0	0	0	0	0	0	0	0	0

0	0	0	0	0	0	0	0	0	0	0
0	0	0	0	0	0	0	0	0	0	0
0	0	0	0	0	0	0	0	0	0	0
0	0	0	0	0	0	0	0	0	0	0
0	0	0	0	0	0	0	0	0	0	0
0	0	0	0	0	0	0	0	0	0	0
0	0	0	0	0	0	0	0	0	0	0
0	0	0	0	0	0	0	0	0	0	0
0	0	0	0	0	0	0	0	0	0	0
0	0	0	0	0	0	0	0	0	0	0
0	0	0	0	0	0	0	0	0	0	0
0	0	0	0	0	0	0	0	0	0	0
0	0	0	0	0	0	0	0	0	0	0
0	0	0	0	0	0	0	0	0	0	0
0	0	0	0	0	0	0	0	0	0	0
0	0	0.1	0.04	0	0.01	0.06	0.01	0.04	0.01	0.01
0	0	0	0	0	0	0	0	0	0	0
0	0	0	0	0	0	0	0	0	0	0
0.01	0	0	0	0	0	0	0	0	0	0
0	0	0	0	0	0	0	0	0	0	0
0	0	0	0	0	0	0	0	0	0	0
0	0	0	0	0	0	0	0	0	0	0
0	0	0	0	0	0	0	0	0	0	0
0	0	0	0	0	0	0	0	0	0	0
0	0	0	0	0	0	0	0	0	0	0
0	0	0	0	0	0	0.01	0	0.01	0	0
0	0	0	0	0	0	0	0	0	0	0
0	0	0	0	0	0	0	0	0	0	0
0	0	0	0	0	0	0	0	0	0	0
0	0	0	0	0	0	0.01	0	0	0	0
0	0	0	0	0	0	0	0	0	0	0
0	0	0	0	0	0	0	0	0	0	0
0.03	0.12	0.05	0.18	0.04	0	0.26	0.01	0.02	0.02	0.01
0	0	0	0	0	0	0	0	0	0	0
0	0	0	0	0	0	0	0	0	0	0
3.21	2.49	0.8	0.36	2.43	1.58	0.73	7.05	0.25	7.35	8.88
0	0	0	0	0	0	0	0	0	0	0
0	0	0	0	0	0	0	0	0	0	0
0	0	0	0	0	0	0	0	0	0	0
0	0	0	0	0	0	0	0	0	0	0
0	0	0	0	0	0	0	0	0	0	0.01
0	0	0	0	0	0	0	0	0	0	0
0.41	0.15	1.05	1.15	0.48	0.73	0.87	0.48	0.95	0.28	0.92
0.02	2.33	0.01	0.02	0.05	0.01	0.1	0.01	0.02	0.01	0.01
0	0	0	0	0	0	0	0	0	0	0
1.9	4.39	2.12	1.77	2.62	4.38	1.14	3.22	3.67	1.68	2.66
0.16	14.9	0.14	0.06	0.22	0.09	0.08	0.09	0.06	0.07	0.07
0	0	0	0	0	0	0	0	0	0	0
0.31	8.27	0.21	0.25	0.69	0.7	0.84	0.18	0.22	0.12	0.21
0	0	0	0	0	0	0	0	0	0	0

0	0	0	0	0	0	0	0	0	0	0
3.78	2.59	2.12	5.04	3.89	2.52	9.68	1.06	4.63	0.65	2.15
0	0	0	0	0	0	0	0	0	0	0
0.09	3.39	0.12	0.13	0.09	0.03	0.27	0.03	0.11	0.03	0.03
0	0.01	0	0	0	0	0	0	0	0	0
0	0.03	0.01	0	0	0	0	0	0	0	0
0	0.04	0	0	0	0	0	0	0	0	0
0	0	0	0	0	0	0	0	0	0	0
0	0.01	0	0	0	0	0	0	0	0	0
0	0.01	0	0	0	0	0	0	0.01	0	0
1.5	1.25	1.95	0.66	0.81	0.88	0.54	0.53	1.97	0.53	1.19
0	0	0	0	0	0	0	0	0	0	0
0.03	0.05	0.02	0.02	0.01	0.06	0.03	0.11	0.1	0.02	0.07
0	0.01	0	0	0	0.02	0	0	0	0	0
0	0.01	0	0	0	0	0	0	0	0	0
0.03	0.07	0.04	0.03	0.06	0.1	0.02	0.01	0.16	0.01	0.09
0.03	0.04	0.15	0.18	0.32	0.64	0.11	0.03	0.23	0.08	0.3
0	0.02	0	0	0.01	0.04	0	0	0	0	0
0	0	0	0	0	0	0	0	0	0	0
0	0.02	0	0	0	0	0	0	0	0	0
0	0	0	0	0	0	0	0	0	0	0
0	0.01	0	0.01	0	0.01	0.07	0	0.01	0	0.03
0.01	2.33	0.01	0.01	0.01	0.01	0.01	0.01	0.01	0	0
0.14	0.08	0.31	0.33	0.31	1.56	0.47	0.22	2.05	0.21	0.85
0	0	0	0	0	0	0	0	0	0	0
0	0	0	0	0	0	0	0	0	0	0
0	0.08	0	0.01	0	0	0.03	0	0.01	0.01	0.01
0	0	0	0	0	0	0	0	0	0	0
0.01	0.05	0.01	0.05	0.01	0.01	0.01	0	0.02	0.01	0
0	0	0	0	0	0	0	0	0	0	0
0.01	0	0.01	0.02	0.09	0.06	0.08	0	0.06	0.01	0.02
0.11	0.03	0.19	0.36	0.1	0.59	0.41	0.11	0.23	0.02	0.06
1.09	10.7	0.49	0.71	0.67	2.52	0.82	0.46	1.74	0.56	1.45
0	0	0	0	0	0	0	0	0	0	0
0	0	0	0	0	0	0	0	0.02	0.02	0
0.11	0.03	0.2	0.44	0.29	1.06	0.25	0.29	0.27	0.32	0.53
0.01	0	0.01	0.01	0	0.01	0	0	0.02	0	0
0.02	0.02	0	0.01	0.01	0.01	0.01	0.01	0.02	0.02	0.9
0.54	0.33	1.18	0.6	0.42	1.91	0.25	0.71	1.47	0.57	0.94
1.13	0.53	0.05	2.28	0.1	0.45	0.13	0.09	0.63	0.07	0.52
0.02	2.76	0.02	0.01	0.06	0.01	0.04	0.05	0.03	0.02	0.02
0.01	1.26	0.03	0.14	0.01	0.01	0.04	0.81	0.04	0.56	0.01
0	0.02	0	0.02	0	0	0.04	0	0	0	0
0.01	0.13	0.02	0	0.03	0.01	0.01	0.18	0.02	0.02	0
0.04	0.6	1.66	0.13	0.07	0.05	0.1	8.96	0.11	9.55	0.06
0.01	0.21	0.02	0.02	0.02	0.01	0.04	2.65	0.02	0.02	0.29
0.33	0.78	0.33	0.7	0.31	0.03	0.46	0.12	2.04	0.23	0.51
0	0.01	0	0	0	0	0	0	0	0	0
0	0	0	0	0	0	0	0	0	0	0
0	0	0	0	0	0.03	0	0	0	0	0

0	0	0	0.01	0	0	0	0	0.01	0	0
0.49	0.07	2.09	0.29	1.23	0.52	0.72	0.27	0.06	0.46	1.66
0	0	0	0	0	0	0	0	0	0	0
0.01	0.4	0.02	0.02	0	0.01	0.01	0.02	0.03	0.01	0.01
0	0	0	0	0	0	0	0	0	0	0
0.01	0.11	0.04	0.02	0	0	0.01	0.21	0.01	0.01	0
0	0	0	0	0	0	0	0	0	0	0
0	0	0	0	0	0	0	0	0	0	0
0.02	0.01	0.02	0.02	0.01	0	0.08	0.01	0.16	0.01	0.01
0	0	0.03	0.02	0.01	0	0.06	0.01	0.02	0	0
0	0	0	0	0	0	0	0	0	0	0
0	0	0	0	0	0	0	0	0	0	0
0	0	0	0	0	0	0	0	0	0	0
0	0.06	0	0	0	0	0	0	0	0	0
0	0	0.01	0.08	0.01	0	0.28	0	0.04	0	0
0	0	0	0	0	0	0	0	0	0	0
0	0.02	0	0	0	0	0	0	0	0	0
0	0	0	0	0	0	0	0	0	0	0
0	0.02	0	0	0	0	0	0	0	0	0
0.01	0.17	0.01	0.11	0.13	0.02	0.04	0.04	0.03	0.03	0.04
0.03	0.23	0.01	0.01	0.02	0	0.01	0.06	0.01	0.03	0.05
0	0	0	0	0	0	0	0	0	0.01	0
0	0	0	0	0	0	0	0	0	0	0
0	0	0	0	0	0	0	0	0	0	0
0	0	0.01	0	0	0	0	0	0	0	0
0	0.22	0	0	0	0	0	0	0	0	0
0	0	0	0	0	0	0	0	0	0	0
0	0.1	0	0	0	0.02	0	0	0.02	0	0
0	0.65	0	0.01	0.01	0.01	0.02	0.08	0	0.01	0
0.03	0.09	0.1	0.23	0.02	0.16	0.06	0.29	0.07	0.13	0.31
0.01	0.3	0.03	0.07	0.01	0.04	0.1	0	0.18	0.02	0.01
0.07	0.01	0.12	0.07	0.53	0.15	0.05	0.01	0.04	0.03	0.14
0	0	0	0	0	0	0	0	0	0	0
25.5	0.83	40.9	22.3	34.7	23.8	21.8	16.5	11.1	41.7	7.53
0	0	0	0	0	0	0	0	0	0	0
0	0	0	0	0	0	0	0	0	0	0
0	0.01	0	0	0	0.02	0	0.02	0	0.02	0
0	0	0	0	0	0	0	0	0	0	0
0	0.02	0	0	0	0	0	0	0	0	0
0	0	0	0	0	0	0	0	0	0	0
0.01	0.07	0	0	0	0	0	0	0.01	0	0
0	0.02	0	0	0	0	0	0	0	0	0
0	0	0	0	0	0	0	0	0	0	0
0	0	0	0	0	0	0	0	0	0	0
0	0	0	0	0	0	0	0	0	0	0
0	0	0	0	0	0	0	0	0	0	0
0	0	0	0	0	0	0	0	0	0	0
0	0	0	0	0	0	0	0	0	0	0
0	0	0	0	0	0	0	0	0	0	0
0	0	0	0	0	0	0	0	0	0	0

0	0	0	0	0	0	0	0	0	0	0
0	0	0	0	0	0	0	0	0	0	0
0	0	0	0	0	0	0	0	0	0	0
0	0	0	0	0	0	0	0	0	0	0
0	0	0	0	0	0	0	0	0	0	0
0	0	0	0	0	0	0	0	0	0	0
0	0	0	0	0	0	0	0	0	0	0
0	0	0	0	0	0	0	0	0	0	0
0	0	0	0	0	0	0	0	0	0	0
0	0	0	0	0	0	0	0	0	0	0
0	0	0	0	0	0	0	0	0	0	0
0	0	0	0	0	0	0	0	0	0	0
0	0	0	0	0	0	0	0	0	0	0
0	0	0	0	0	0	0	0	0	0	0
0	0	0	0	0	0	0	0	0	0	0
0	0	0	0	0	0	0	0	0	0	0
0	0	0	0	0	0	0	0	0	0	0
0	0	0	0	0	0	0	0	0	0	0
0	0	0	0	0	0	0	0	0	0	0
0	0	0	0.01	0	0	0	0	0	0	0
0	0	0	0	0	0	0	0	0	0	0
0	0	0	0	0	0	0	0	0	0	0
0	0	0	0	0	0	0	0	0	0	0
0	0	0	0	0	0	0	0	0	0	0
0	0	0	0	0	0	0	0	0	0	0
0	0	0	0	0	0	0	0	0	0	0
0	0	0	0	0	0	0	0	0	0	0
0	0	0	0	0	0	0	0	0	0	0
0	0	0	0	0	0	0	0	0	0	0
0	0	0	0	0	0	0	0	0	0	0
0	0	0	0	0	0	0	0	0	0	0
0	0	0	0	0	0	0	0	0	0	0
0	0	0	0	0	0	0	0	0	0	0
0	0	0	0	0	0	0	0	0	0	0
0	0	0	0	0	0	0	0	0	0	0
0	0	0	0	0	0	0	0	0	0	0
0	0	0	0	0	0	0	0	0	0	0
0	0	0.02	0.01	0	0	0	0	0	0	0
0	0	0.01	0.01	0	0	0	0	0	0	0
0	0	0	0	0	0	0	0	0.01	0	0
0	0	0	0	0	0	0	0	0	0	0
0	0	0	0	0	0	0	0	0	0	0
0	0	0	0	0	0	0	0	0	0	0
0	0	0	0	0	0	0	0	0	0	0
0.1	0.09	0.89	2.14	0.13	0.74	0.29	1.05	0.04	2.51	0.09
0	0	0	0	0	0	0	0	0	0	0
0	0	0	0	0	0	0	0	0	0	0
0	0	0	0	0	0	0	0	0	0	0
1.69	0.04	2.05	0.36	0.03	0.03	0.21	0.04	0.06	0.13	0.04
0	0	0	0	0	0	0	0	0	0	0

0	0	0	0	0	0	0	0	0	0	0
0	0	0	0	0	0	0	0	0	0	0
0.01	0	0.01	0	0	0	0	0	0	0	0
0.01	0	0.01	0	0	0	0	0	0	0	0
0.17	0	0.16	0.03	0	0	0.02	0	0	0.01	0
0	0	0	0	0	0	0	0	0	0	0
0	0	0	0	0	0	0	0	0	0	0
0.01	0	0.01	0	0	0	0	0	0	0	0
0.6	0.01	0.64	0.11	0	0.01	0.06	0.01	0.01	0.04	0.01
0	0	0	0	0	0	0	0	0	0	0
0.01	0	0.01	0	0	0	0	0	0	0	0
0	0	0	0	0	0	0	0	0	0	0
0	0	0	0	0	0	0	0	0	0	0
0	0	0	0	0	0	0	0	0	0	0
0	0	0	0	0	0	0	0	0	0	0
0	0.01	0	0	0	0	0	0	0	0	0
0	0	0	0	0	0	0	0	0	0	0
0	0	0	0	0	0	0	0	0	0	0
0	0	0	0	0	0	0	0	0	0	0
0	0	0	0	0	0	0	0	0	0	0
0	0	0	0	0	0	0	0	0	0	0
0	0	0	0	0	0	0	0	0	0	0
0	0	0	0	0	0	0	0	0	0	0
0	0	0	0	0	0	0	0	0	0	0
0	0	0	0	0	0	0	0	0	0	0
0.02	0	0	0	0	0	0	0	0	0	0
0	0	0	0	0	0	0	0	0	0	0
0	0	0	0	0	0	0	0	0	0	0
0	0	0	0	0	0	0	0	0	0	0
0	0	0	0	0	0	0	0	0	0	0
0	0	0	0	0	0	0	0	0	0	0
0	0	0	0	0	0	0	0	0	0	0
0	0	0	0	0	0	0	0	0	0	0
0	0	0	0	0	0	0	0	0	0	0
0	0	0	0	0	0	0	0	0	0	0
0	0	0	0	0	0	0	0	0	0	0
0	0	0	0	0	0	0.19	0	0	0	0
0	0.03	0.05	0	0.01	0	0.01	0.01	0	0.02	0.1
0.08	0.01	0.26	0.12	0.13	0.01	0.03	0.01	0.45	0.04	0.04
0.95	0.52	1.21	0.59	0.56	0.12	1.1	0.58	1.13	0.62	1.3
0.01	0.01	0	0	0	0.02	0	0	0.07	0.01	0
0	0	0	0	0	0	0	0	0	0	0
0	0	0	0	0	0	0	0	0	0	0
0	0	0	0	0	0	0	0	0	0	0
0.29	0.15	0.13	0.77	0.83	0.65	1.92	0.09	1.75	0.13	0.13
0	0	0	0	0	0	0	0	0	0	0
0	0	0	0	0	0	0	0	0	0	0
0	0.02	0	0	0	0	0.07	0.02	0.01	0	0.01
0	0	0	0.02	0	0	0	0	0.02	0	0
0	0.55	0	0	0	0.03	0	0	0	0	0

0	0	0	0	0	0.01	0.02	0.01	0	0	0
0	0.01	0	0	0	0.02	0	0	0	0	0
0	0	0	0	0	0	0	0	0	0	0
0.01	2.18	0.01	0.01	0.02	0.02	0.01	0.01	0.02	0.02	0.01
0	0	0	0	0	0	0	0	0	0	0
0	0	0	0	0	0	0	0	0	0	0
0	0	0	0	0	0	0	0	0	0	0
0	0	0	0	0	0	0	0	0	0	0
0	0	0	0	0	0	0	0	0	0	0
2.36	2.07	5.02	9.01	1.33	0.24	7.48	0.51	2.01	0.43	0.15
0	0	0	0	0	0	0	0	0	0	0
0	0	0	0	0	0	0	0	0	0	0
0	0	0	0	0	0	0	0	0	0	0
0	0.01	0	0.01	0	0	0	0	0	0	0
0	0.01	0.02	0.08	0	0	0.04	0	0.02	0	0
0	0	0	0	0	0	0	0	0	0	0
0	0	0	0	0	0	0	0	0	0	0
0	0	0	0	0	0	0	0	0	0	0
0	0	0	0	0	0	0	0	0	0	0
0	0	0	0	0	0	0	0	0	0	0
0	0	0	0	0	0.01	0.01	0	0	0	0
0	0	0	0	0	0	0	0	0	0	0
0	0	0	0	0	0	0	0	0	0	0
0	0	0	0	0	0	0	0	0	0	0
0.01	0.01	0.01	0.02	0	0	0.02	0	0.01	0	0
0	0	0	0	0	0	0	0	0	0	0
0	0	0	0	0	0	0	0	0	0	0
0	0	0	0	0	0	0	0	0	0	0
0	0	0	0	0	0	0	0	0	0	0
0	0	0	0	0	0	0	0	0	0	0
0	0	0	0	0	0	0	0	0	0	0
0	0	0	0	0	0	0	0	0	0	0
0.1	0.01	0	0.01	0.03	0.05	0.08	0.02	0.01	0.01	0.01
0.72	0.02	0.01	0.04	0.4	0.81	0.26	0.08	0.1	0.03	0.08
0.02	0	0	0	0.01	0	0.01	0	0.02	0	0
0	0	0	0	0	0	0	0	0	0	0
0	0	0	0	0	0	0	0	0	0	0
5.19	0.08	0.07	0.15	2.73	1.84	3.09	0.94	1.06	0.15	0.26
0	0	0	0	0	0	0	0	0	0	0
1.09	0.02	0.09	0.03	0.67	0.25	0.02	0.14	0.01	0.08	0.15
0	0	0	0	0	0	0	0	0	0	0
0	0	0	0	0.01	0	0.01	0	0.01	0	0
0	0	0	0	0	0	0	0	0	0	0
0.04	0	0.07	0.01	0.05	0.06	0.04	0.01	0.08	0.01	0.09
0	0	0	0	0	0	0	0	0	0	0
0	0	0	0	0	0	0	0	0	0	0
0	0	0	0	0	0	0	0	0	0	0
0	0	0	0	0	0	0	0	0	0	0
0	0	0	0	0	0	0	0	0	0	0
0	0	0	0	0	0	0	0	0	0	0

0	0	0	0	0	0	0	0	0	0	0
0	0	0	0	0	0	0	0	0	0	0
0	0	0	0	0	0	0	0	0	0	0
0	0	0	0	0	0	0	0	0	0	0
0	0	0	0	0	0	0	0	0	0	0
0	0	0.02	0	0	0	0	0	0	0	0
0	0	0	0	0	0.03	0	0	0	0	0
0	0	0	0	0	0	0	0	0	0	0
0	0.08	0.02	0	0	0.01	0	0	0.01	0	0
0	0	0	0	0	0	0	0	0	0	0
0.02	3.24	0.01	0.01	0.15	0.08	0.01	0.01	0.02	0.01	0.01
0	0	0	0	0	0	0	0	0	0	0
0	0	0	0	0	0	0	0.22	0	0	0
0	0.02	0	0	0	0	0	0.01	0	0	0
0	0	0	0	0	0	0	0	0	0	0
0	0.01	0	0	0	0	0	0	0	0	0
0	0	0	0	0	0	0	0	0	0	0
0	0	0	0	0	0	0	0	0	0	0
0	0	0.02	0	0	0	0	0	0	0	0
0	0.02	0	0	0	0.01	0	0	0	0	0
0	0	0	0	0	0	0	0	0	0	0
0	0.01	0	0	0	0	0	0	0	0	0
0	0	0	0	0	0	0	0	0	0	0
0	0	0	0	0	0.01	0	0	0	0	0
0.02	2.34	0.04	0.07	0.08	0.05	0.06	0.01	0.02	0.04	0.03
0	0.01	0	0	0	0.01	0	0	0	0	0
0	0	0	0	0	0	0	0	0	0	0
0	0	0	0	0	0	0	0	0	0	0
0	0.23	0	0	0.02	0.03	0.01	0	0	0	0
0	0	0	0	0	0	0	0	0	0	0
0	0	0	0	0	0	0	0	0	0	0
0.02	0	0	0	0	0	0	0	0	0	0
0	0	0	0	0	0.01	0	0	0	0	0
0	0	0	0	0	0	0	0	0	0	0
